# Financial accessibility of school meals: evidence from parental willingness to co-pay in the UK

**DOI:** 10.3389/fpubh.2026.1839749

**Published:** 2026-06-11

**Authors:** Bisola Osifowora, Owen Nicholas, Raymond Oppong, Viktorija Kesaite, Amy Yau, Irina Pokhilenko, Martin White, Emma Frew

**Affiliations:** 1 Health Economics Unit, School of Health Sciences, College of Medicine and Health, University of Birmingham, Birmingham, United Kingdom; 2 Department of Statistical Sciences, University College London, London, United Kingdom; 3 MRC Epidemiology Unit, School of Clinical Medicine, University of Cambridge, Cambridge, United Kingdom; 4 Population Health Innovation Lab, Department of Public Health, Environments and Society, Faculty of Public Health and Policy, London School of Hygiene and Tropical Medicine, London, United Kingdom

**Keywords:** child nutrition, contingent valuation, co-payment, food insecurity, health inequalities, public health policy, school meals, willingness to co-pay

## Abstract

**Background:**

School meals are an established population-level intervention to improve dietary intake and reduce health inequalities, yet expanding access is constrained by affordability barriers. This study aims to assess parental willingness to contribute to a subsidised co-payment model for primary school meals and to identify the socioeconomic, behavioral, and food insecurity-related factors influencing participation.

**Methods:**

A cross-sectional survey of 1,009 UK parents was conducted using a contingent valuation approach. Parents were asked about their willingness to contribute toward both a standard and nutritionally enhanced school meal. Multivariable regression analyses were used to examine associations between willingness to pay (WTP) and socioeconomic, behavioral, and food insecurity-related factors.

**Results:**

Eighty-eight percent of parents indicated a willingness to contribute to a subsidised school meal scheme. Among those willing to contribute, the mean willingness to pay was £2.45 per meal, with higher contributions observed for nutritionally enhanced meal options. Participation decreased as costs increased, indicating affordability as a key determinant of uptake. Socioeconomic factors, food insecurity, and prior engagement with school-based food provision were significantly associated with both willingness to pay and contribution levels. There was also evidence that parents were willing to contribute more toward nutritionally enhanced meal options.

**Conclusion:**

These findings highlight the importance of affordability and equity in the design of co-payment models for school meals. The results provide policy-relevant evidence to inform the development of financially sustainable and inclusive school meal programmes, including consideration of how nutritional quality influences parental engagement and perceived value.

## 1 Introduction

Poor diet quality is a leading modifiable risk factor for non-communicable diseases globally, and the burden falls disproportionately on children from lower-income households (1, 2). Worldwide, an estimated 181 million children under five are living in severe child food poverty (3), with school-aged children increasingly facing diet-related health challenges such as childhood overweight and obesity alongside persistent micronutrient deficiencies as reported by the World Health Organisation (WHO) (4). In the United Kingdom, one in three children leaving primary school are classified as overweight or obese (5) and dietary quality among school-aged children remains suboptimal, with excessive intakes of free sugars, saturated fat, and sodium and inadequate consumption of fruit, vegetables, and fibre (6). These dietary patterns are shaped by socioeconomic circumstances, and children experiencing food insecurity are at particular risk of poor nutritional outcomes (7). In 2024, an estimated 2.7 million children in the UK lived in households experiencing food insecurity (8), a challenge shared across high income countries. In the United States, nearly 14 million children experienced household food insecurity in 2023 (9), while Canada had approximately 2.5million children, roughly one in three being affected in 2024 (10, 11). This has significant implications for a child’s physical health, cognitive development, and educational attainment. Improving dietary intake in childhood is therefore a central objective of public health policy, given its implications for lifelong health trajectories and non-communicable disease prevention.

School meals represent one of the most direct population-level mechanisms available to governments to improve children’s dietary intake and narrow diet-related health inequalities. A growing body of international evidence demonstrates that access to school meals is associated with improved diet quality, reduced consumption of foods high in fat, salt, and sugar, reductions in childhood overweight, improved educational attainment, and financial savings for households (7, 12–18). In England, the Universal Infant Free School Meal (UIFSM) programme, which since 2014 has provided free lunches to all children in reception and years 1–2 (ages 4–7), has led to measurable reductions in consumption of unhealthy foods, lower pupil body weight, and household expenditure savings (19, 20). These findings are consistent with the wider international literature establishing school meal provision as both a public health and an anti-poverty intervention (15). Beyond the infant years, school meals in England are provided to older primary school pupils either through means-tested free school meals (FSMs) (17) or at full cost to households.

Expansion of the FSM provision to all pupils has been an important topic on the UK political agenda for the past few years (21, 22); however, due to the perceived high cost and the uncertainty that a ‘one size fits all’ policy will be politically acceptable, it has not been implemented. There have been calls for universal FSM expansion (22) as a priority public health policy, and as a result, the government has recently announced that from September 2026, all children in families receiving Universal Credit will become eligible for FSMs (23, 24). While this represents a significant step, many children remain ineligible, particularly those in households not in receipt of universal credit. These children, who may still face dietary and financial pressures, are effectively excluded from a proven public health intervention.

Scaling universal free school meal provision to all primary school children carries substantial fiscal implications. In this context, a subsidised co-payment model, in which households currently ineligible for FSMs contribute a portion of the meal cost while the government covers the remainder may offer a pragmatic pathway to expanding coverage but raises important questions about financial accessibility and potential impacts on health inequalities. Such a model could extend the reach of school meals as a dietary improvement intervention while containing public expenditure. To inform the design of such a policy, it is essential to understand how much households would be willing to contribute, and which factors influence their willingness.

This study used the contingent valuation method to estimate the maximum willingness to pay (WTP) of parents of UK primary school children who are currently ineligible for FSMs. We focused on primary schools because parents are the key decision-makers regarding their child’s meal arrangements at this stage, whereas in secondary schools’ adolescents exercise greater autonomy over food choices, and the organisation of school food provision differs substantially (25). Despite growing policy interest in expanding school meal provision, there is currently no UK evidence on whether parents would be willing to contribute toward a subsidised co-payment model, or on the factors that would influence participation and uptake. This evidence is essential for designing financially sustainable and equitable school meal schemes that maximize coverage without widening health inequalities. This study therefore had two objectives: first, to estimate the maximum WTP for a standard school lunch (assuming no change to current perceived quality); and second, to estimate the maximum WTP for a school lunch described as nutritious and of high quality. We also examined the socioeconomic, behavioral, and food insecurity-related factors associated with both participation and contribution levels, with a focus on identifying potential barriers to equitable access.

## 2 Methods

### 2.1 Survey design and administration

This was a cross-sectional online survey of parents and guardians of UK primary school children who were not eligible for FSMs. The survey instrument was designed for self-completion and administered by The Food Foundation (8) through YouGov, a global online survey provider that maintains a panel of over 2 million UK adults recruited through a variety of channels (26). The survey was piloted and revised with the study team and a public advisory group based at the University of Birmingham prior to deployment. The final survey was open from 25th March to 12th April 2024 and sampled a total of 1,009 parents and guardians. Full details of the survey instrument are presented in Supplementary File 1.

WTP was elicited using the contingent valuation method, a stated-preference technique originally developed in environmental economics to value non-market goods and widely applied in health economics to estimate public valuations of health-related interventions (27, 28). In public health research, contingent valuation is used to assess acceptability and accessibility of interventions where real-world implementation data are not available. Respondents were first provided with a description of the current FSM policy and the proposed co-payment scheme. The following scenario description was developed by the research team, informed by the existing FSM policy framework and the proposed co-payment model under investigation, which was then refined through pilot testing with a public advisory group based at the University of Birmingham.

*“For pupils who are currently not eligible for a FSM, instead of asking households to pay the full cost for a school meal, we explore a subsidised alternative whereby households part-pay, and the government subsidise the remaining cost. Only households who are currently not eligible for a FSM would be given the opportunity to contribute”*.

Respondents were then asked whether they would opt for a school lunch (instead of a packed lunch) and to indicate their maximum WTP on a bounded open-ended scale from £0 (unwilling to contribute) to £5. Respondents were informed that the average cost of a school lunch in England is approximately £2.70 but that their WTP could be higher or lower than this amount. WTP was elicited twice: first for a standard school lunch (assuming no change to current perceived quality), and second for a school lunch described as nutritious and of high quality. No specific definition of ‘nutritious and of high quality’ was provided; respondents interpreted this based on their own understanding of nutritional quality.

Following the WTP questions, the survey collected data on sociodemographic characteristics, economic and employment status, perceptions of school lunch quality, children’s current lunch arrangements, breakfast club attendance, and food insecurity status.

### 2.2 Participants and sampling

The target population comprised parents and guardians of primary school children (ages 4–11) in the UK who were not eligible for FSMs. YouGov sampled respondents from its online panel using active sampling to achieve a sample broadly representative of the target population in terms of age, gender and geographic region.

### 2.3 Conceptual framework

**Figure 1:**
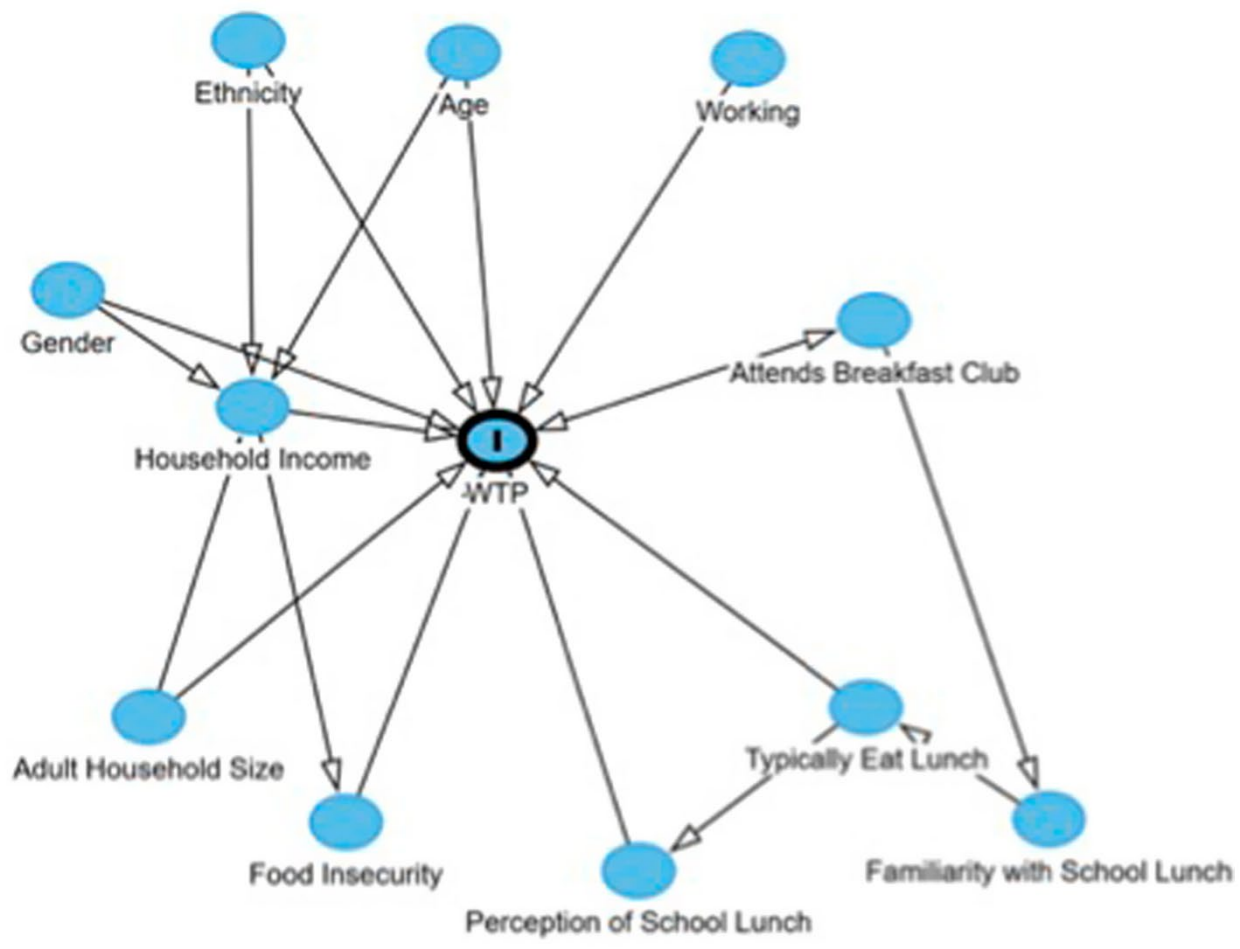
Directed acyclic graph.

A directed acyclic graph (DAG) was developed using DAGitty (version 3.0) to map the hypothesized relationships between sociodemographic, economic, behavioral factors and WTP (Figure 1). The DAG served as a conceptual framework guiding the selection of covariates for regression modeling, rather than as a formal causal identification strategy, given the cross-sectional study design. The framework reflects pathways through which socioeconomic and behavioral factors may influence participation in the subsidised school meal programme. All covariates included in the models were pre-specified based on the DAG and prior literature.

Three hypotheses were formulated *a priori*:

*H1*: Parents will be WTP more for a nutritious school lunch than for a standard school lunch.

*H2*: Households with higher income will express a higher WTP, reflecting greater ability to pay.

*H3*: Greater engagement with school food (e.g., higher frequency of school lunch consumption, more positive perceptions of school lunch quality) will be associated with higher WTP.

### 2.4 Variables

#### 2.4.1 Outcome variables

Two sets of outcomes were modeled. First, the binary decision not to contribute (coded 1 = not willing to pay, 0 = willing to pay) was analysed to identify predictors of non-participation. Second, among those expressing a positive WTP, the continuous WTP amount (in £) was modelled separately for the standard and nutritious school lunch scenarios.

#### 2.4.2 Explanatory variables

Sociodemographic variables included gender (male [reference], female), age (midpoint of reported age range, treated as continuous), working status (No [reference], Yes), and adult household size (number of adults aged 16 + years, continuous). Ethnicity was coded as a binary variable (White [reference], ethnic minority). Gross annual household income was converted from reported income bands to a midpoint-based continuous measure expressed in units of £10,000 (e.g., “£70,000–£99,999 per year” = 8.5). For the highest open-ended income band, the lower bound was used as a conservative estimate.

Behavioral engagement variables captured children’s typical lunch arrangements (school lunch [reference], packed lunch, or mixed), parental familiarity with the school menu (no (reference), yes), and parental perception of school lunch quality (high [reference], medium, low). Breakfast club attendance was recorded as a binary variable.

Food insecurity was measured using four binary indicators adapted from the United States Department of Agriculture’s Food Security Survey Module, a validated instrument widely used in high-income countries including the UK (7). This was self- reported and respondents indicated whether, in the past month and in the past 12 months, they had experienced: (i) smaller meals than usual, (ii) hunger without eating due to unaffordability, (iii) not eating for a whole day, or (iv) none of these. Responses were summed to create a food insecurity severity index (0 = food secure; 1 = mild; 2 = moderate; 3 = severe; 4 = very severe), constructed separately for the past-month and past-year reference periods. A full summary of all variables, their definitions and theoretical justifications for inclusion is provided in Supplementary File 2.

### 2.5 Demand curves

Demand curves were constructed by calculating the cumulative proportion of respondents WTP at or above each £0.25 price intervals from £0 to £5, separately for the standard and nutritious lunch scenarios. 95% confidence intervals were estimated using the binomial standard error for proportions.

### 2.6 Statistical analysis

All analyses were conducted in R (version 4.5.1, June 2025). A two-stage analytical approach, commonly applied in contingent valuation studies, was used because the data contained two distinct decision processes: first, whether to participate at all (a binary decision), and second, how much to contribute (a continuous amount conditional on participation) (29). These questions require different statistical methods, a logistic regression to identify barriers to WTP, and linear regression (estimated by ordinary least squares (OLS)) to examine factors associated with positive WTP amounts (29).

#### 2.6.1 Logistic regression

Logistic regression was used to model barriers to WTP (NotWTP = 1). This coding was chosen as understanding barriers to participation is of primary interest for policy design. Results are reported as odds ratios (ORs) with standard errors and level of significance. Model fit was assessed using the Akaike Information Criterion (AIC), and standard diagnostic checks confirmed no multicollinearity (variance inflation factors < 2) and no perfect separation. Formal model specifications are presented in Supplementary File 4.

#### 2.6.2 Linear regression

Among the 892 parents expressing a positive WTP, linear regression was used to model the WTP amount for both the standard and the nutritious school lunch. Standard regression models with individual-level covariates were estimated. Formal model specifications and model diagnostics are presented in Supplementary File 4. Mild heteroskedasticity, detected by the Breusch–Pagan test, was addressed using heteroskedasticity-consistent (HC1) robust standard errors (30–32). Residual diagnostics, Durbin–Watson statistics, and variance inflation factors confirmed that key OLS assumptions were met.

### 2.7 Interaction effects

Several theoretically plausible two-way interactions (income × household size, perception of school lunch quality × typical lunch arrangement, gender × income) were tested. Interaction terms were retained in the final model only when statistically significant (*p* < 0.05) and when they improved model fit as assessed by AIC.

### 2.8 Sensitivity analysis

WTP values were bounded between £0 and £5, hence Tobit regression models were estimated as sensitivity analyses to test for potential censoring bias. Tobit models account for the possibility that latent WTP values may extend beyond the observed bounds. Interaction effects consistent with the main models were also tested within the Tobit specifications. Results are reported in Supplementary File 7.

## 3 Results

### 3.1 Sample characteristics

Table 1 presents the sample characteristics. The sample comprised 1,009 parents with a mean age of 39.5 years and a median household income of £55,000. Just over half were female, and the large majority identified as White. Most households reported being food secure in the past month, and approximately two-fifths of children ate school lunches daily. Full descriptive statistics are reported in Table 1.

**Table 1:** Descriptive statistics of survey respondents (*N* = 1,009).

Panel A. respondent characteristics—continuous variables				
Variable	*N*	Mean	SD	Median
Age of parents (years)	984	39.5	6.7	39.5
Gross household annual income (units of £10,000)	880	7.00	3.66	5.50
Adult household size (aged over 16)	1,009	2.02	0.48	2.00

Panel B. Respondent characteristics—categorical variables	
Variable	*n* = 1,009 (%)
Sex	
Male	469 (46.5)
Female	540 (53.5)
Child attends school breakfast club	
Yes	266 (27.3)
No	708 (72.7)
Do you pay for the breakfast club?	
Yes	186 (69.9)
No, the school provides the club free	80 (30.1)
How often do you pay for the breakfast club?	
Daily	67 (37.0)
Weekly	38 (21.0)
Monthly	54 (29.8)
At the start of each half term, each term	22 (12.2)
Child typically eats school lunch	
Yes, they eat lunch provided by the school every day	399 (39.5)
No, they bring a packed lunch from home every day	313 (31.0)
Mixed, combination of school and packed lunch	297 (29.4)
Familiarity with school lunch menu	
Yes	865 (85.7)
No	144 (14.3)
Perception of current offer	
High (Lots of choice and variety)	130 (15.0)
Medium (Average choice and variety)	572 (66.1)
Low (Not much choice and variety)	163 (18.8)
Are you working?	
Yes	908 (90.0)
No	101 (10.0)
Ethnicity	
White	843 (89.5)
Ethnic minority	99 (10.5)

Food insecurity index	In last month	Last 12 months
Food secure	844 (83.7)	805 (79.8)
Mild food insecurity	0 (0.0)	68 (6.7)
Moderate food insecurity	138 (13.7)	70 (6.9)
Severe food insecurity	16 (1.6)	64 (6.3)
Very severe food insecurity	11 (1.1)	2 (0.2)

Panel C. Willingness-to-pay (WTP) outcomes				
Are you willing to contribute toward a school lunch?				*n* = 1,009 (%)
Yes	892 (88.4)			892 (88.4)
No	117 (11.6)			117 (11.6)
	*N*	Mean (£)	SD (£)	Median (£)
Amount WTP toward a standard school lunch	892	2.03	0.97	2.00
Amount WTP toward a nutritious school lunch	892	2.37	1.06	2.00

### 3.2 Demand curves

**Figure 2:**
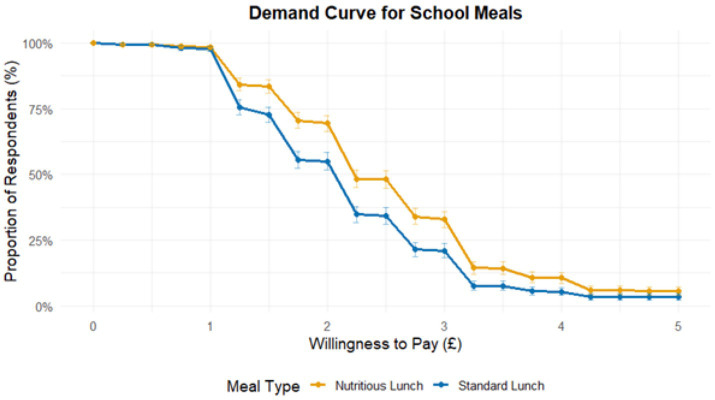
Demand curve for school meals.

Both demand curves exhibited a clear downward slope: as the hypothetical co-payment price increased, the proportion of parents willing to pay decreased (Figure 2). At all price points, the curve for the nutritious school lunch lay above that for the standard lunch. For the standard lunch, approximately 55% of parents were WTP at least £2.00, falling to approximately 20% at £3.00. For the nutritious lunch, approximately 75% were WTP at least £2.00, declining to approximately 35% at £3.00.

### 3.3 Overall willingness to co-pay

**Figure 3:**
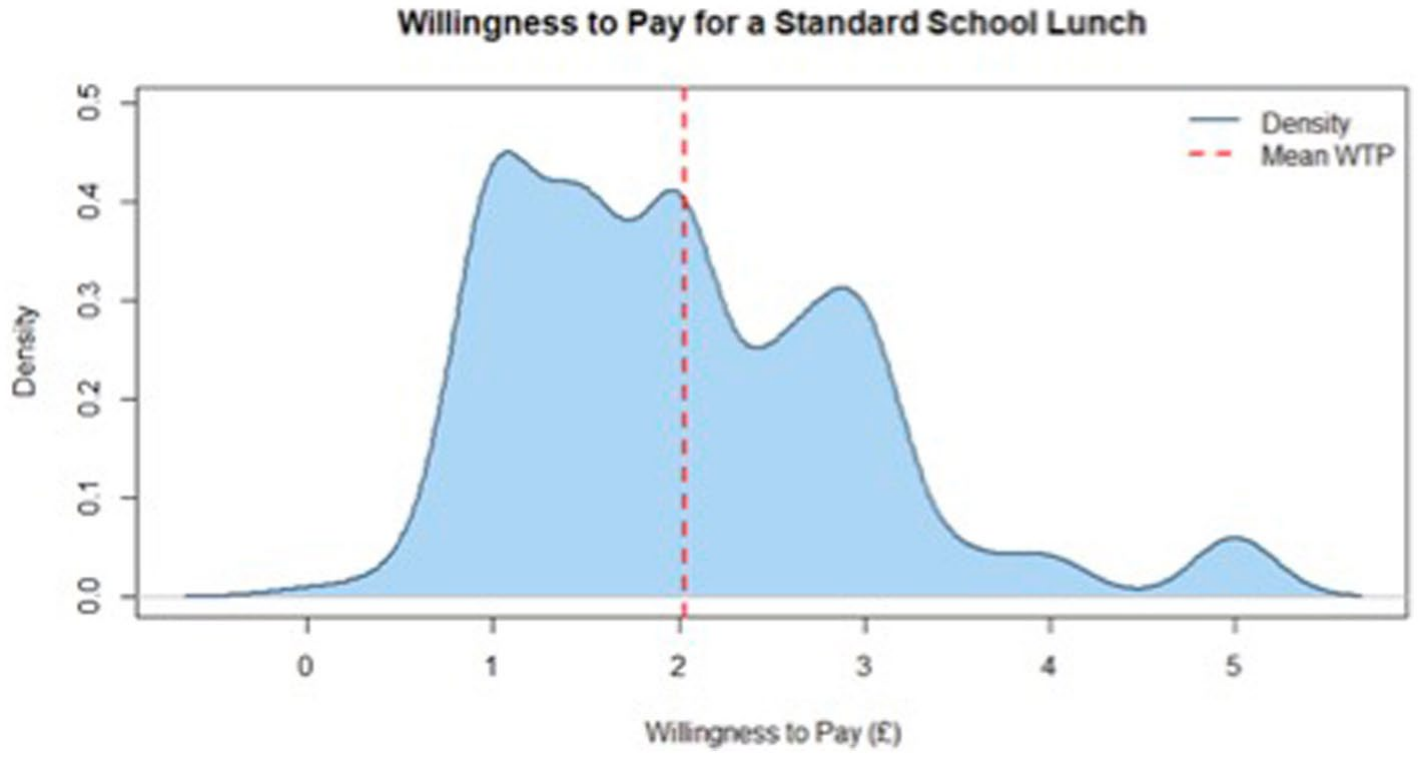
Kernel-density estimate of parents’ willingness to pay (WTP) for a standard school lunch (£). The red dashed line indicates the mean WTP (£2.03).

**Figure 4:**
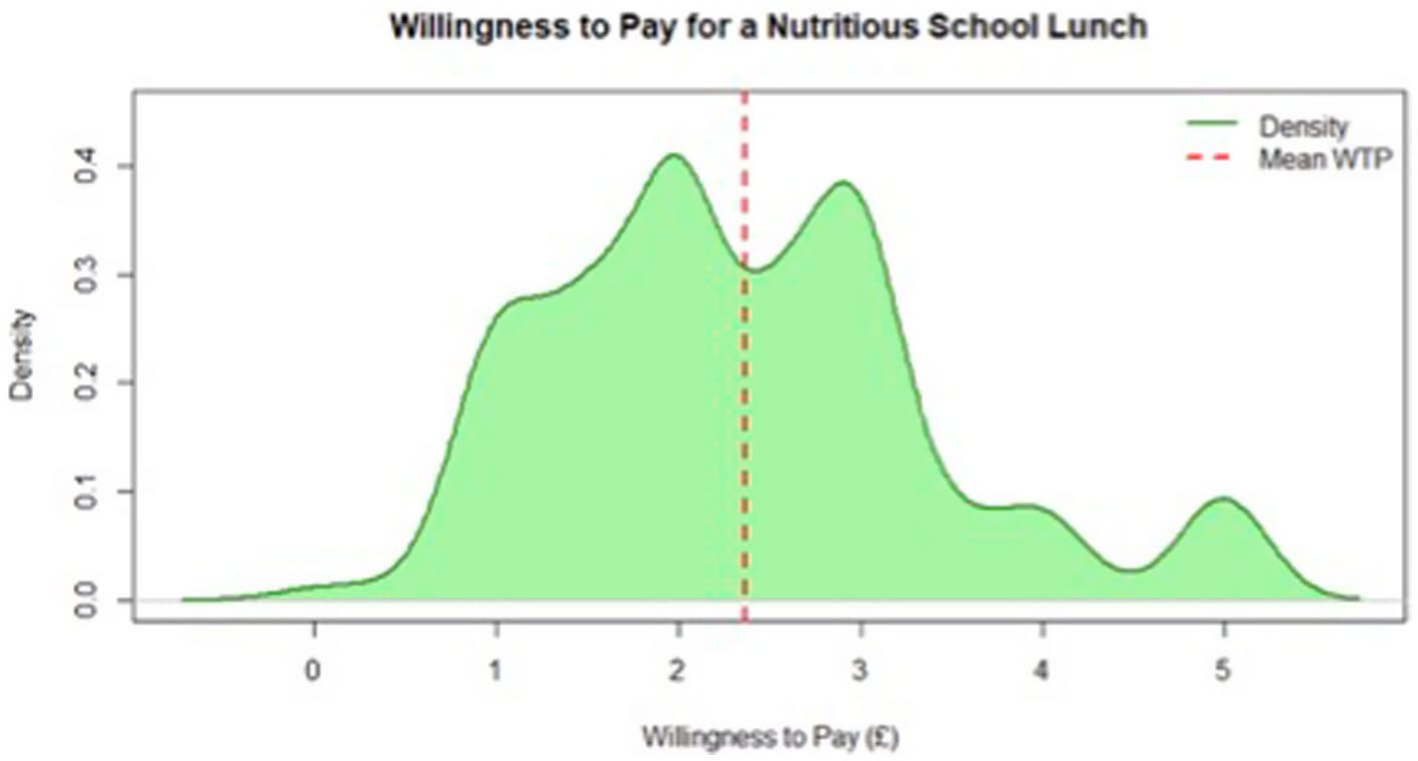
Kernel-density estimate of parents’ willingness to pay (WTP) for a nutritious school lunch (£). The red dashed line indicates the mean WTP (£2.37).

Of the 1,009 respondents, 892 (88.4%) expressed a WTP toward a school lunch, while 117 (11.6%) were unwilling to pay at any price. For the standard school lunch, the mean observed WTP was £2.03 (SD = 0.97, median = £2.00) as shown in Figure 3. For the nutritious school lunch, the mean observed WTP was £2.37 (SD = 1.07, median = £2.00) as shown in Figure 4.

### 3.4 Barriers to WTP for a school lunch

Table 2 presents the results from the final logistic regression model. Intermediate model specifications are reported in Supplementary Table S2. Higher household income, very severe food insecurity in the last month, and breakfast club attendance were all significantly associated with greater odds of being unwilling to pay. There was also a statistically significant interaction between gender and income.

**Table 2:** Factors associated with not being willing to contribute toward the cost of a school lunch.

Barriers to parents’ willingness to co-pay for school lunches	
Dependent variable: unwilling to co-pay (1 = No, 0 = Yes)	
Variable	Final model
Female	1.99 (0.96)
Age (years)	0.91*** (0.03)
Income	1.23** (0.07)
Adult household size	1.08 (0.38)
Moderate food insecurity (last month)	0.98 (1.39)
Severe food insecurity (last month)	10.51 (1.72)
Very severe food insecurity (last month)	23.08* (1.51)
Mild food insecurity (12-month)	<0.001 (856.26)
Moderate food insecurity (12-month)	0.85 (0.98)
Severe food insecurity (12-month)	0.89 (1.55)
Very severe food insecurity (12-month)	<0.001 (4,571.12)
Perception of school lunch (Medium)	0.44 (0.45)
Perception of school lunch (Low)	0.96* (0.56)
Typically eat lunch (No)	1.60 (0.51)
Typically eat lunch (Mixed)	1.39 (0.46)
Attends school breakfast club	2.69** (0.38)
Working	2.66 (1.00)
Ethnicity (Ethnic minority)	1.06 (0.59)
Female × Income	0.76* (0.13)
Constant	0.28 (1.80)
Observations	652
Log likelihood	−119.52
Akaike Inf. Crit.	279.04

***p < 0.001; **p < 0.01; ^*^p < 0.05; *p* < 0.10. Robust (HC1) standard errors in parentheses.

### 3.5 Predicting the maximum amount and factors associated with a positive WTP for a school lunch (standard and of a high nutritional quality)

#### 3.5.1 WTP for a standard school lunch

Table 3 presents the regression results. Lower WTP was significantly associated with being female, older age, moderate food insecurity in the last 12 months, lower perceptions of school lunch quality, and mixed school lunch arrangements (alternating between school and packed lunches). In contrast, a higher household income and larger adult household size were significantly associated with higher WTP. Significant interactions were also observed between income and household size, and between lunch quality perceptions and mixed lunch arrangements.

**Table 3:** Presents the final regression models for the WTP for a school lunch (Standard and high nutritional quality results).

Dependent variable: WTP (£) for a school lunch		
Variable	Standard lunch	Nutritious lunch
Female	−0.25** (0.08)	−0.21* (0.09)
Age (years)	−0.02** (0.01)	−0.01 (0.01)
Income	0.17*** (0.04)	0.05*** (0.01)
Adult household size	0.36** (0.13)	−0.01 (0.07)
Moderate food insecurity (last month)	0.12 (0.22)	0.24 (0.28)
Severe food insecurity (last month)	−0.01 (0.31)	−0.21 (0.36)
Very severe food insecurity (last month)	0.21 (0.21)	0.08 (0.37)
Mild food insecurity (12 months)	−0.37 (0.25)	−0.49 (0.30)
Moderate food insecurity (12 months)	−0.43** (0.14)	−0.55*** (0.16)
Severe food insecurity (12 months)	−0.33 (0.25)	−0.59 (0.31)
Very severe food insecurity (12 months)	−0.25 (0.70)	−0.62 (0.69)
Perception of school lunch (Medium)	−0.43* (0.21)	−0.13 (0.14)
Perception of school lunch (Low)	−0.78** (0.25)	−0.26 (0.16)
Typically eats school lunch (No)	−0.20 (0.30)	−0.48*** (0.11)
Typically eats school lunch (Mixed)	−0.63* (0.31)	−0.38*** (0.10)
Attends school breakfast club	0.07 (0.10)	0.13 (0.11)
Working	0.14 (0.12)	0.15 (0.15)
Ethnicity (Ethnic minority)	−0.24 (0.14)	−0.34* (0.13)
Income × Adult household size	−0.07*** (0.02)	
Perception (Medium) × Typically eat lunch (No)	−0.12 (0.32)	
Perception (Low) × Typically eat lunch (No)	−0.05 (0.35)	
Perception (Medium) × Typically eat lunch (Mixed)	0.43 (0.32)	
Perception (Low) × Typically eat lunch (Mixed)	0.80* (0.36)	
Constant	2.41*** (0.42)	2.98*** (0.39)
Observations	609	609
*R* ^2^	0.18	0.19
Adjusted *R*^2^	0.15	0.16
AIC	1591.84	1707.60

***p < 0.001; **p < 0.01; ^*^p < 0.05; *p* < 0.10. Robust (HC1) standard errors in parentheses for both models.

#### 3.5.2 WTP for a nutritious school lunch

For the nutritious school lunch, being female, moderate food insecurity in the last 12 months, typically bringing packed lunches, mixed lunch arrangements and an ethnic minority status were all significantly associated with lower WTP. Higher household income was significantly associated with higher WTP. Alternative model specifications and their performance metrics are reported in Supplementary File 6.

### 3.6 Predicted WTP

**Figure 5:**
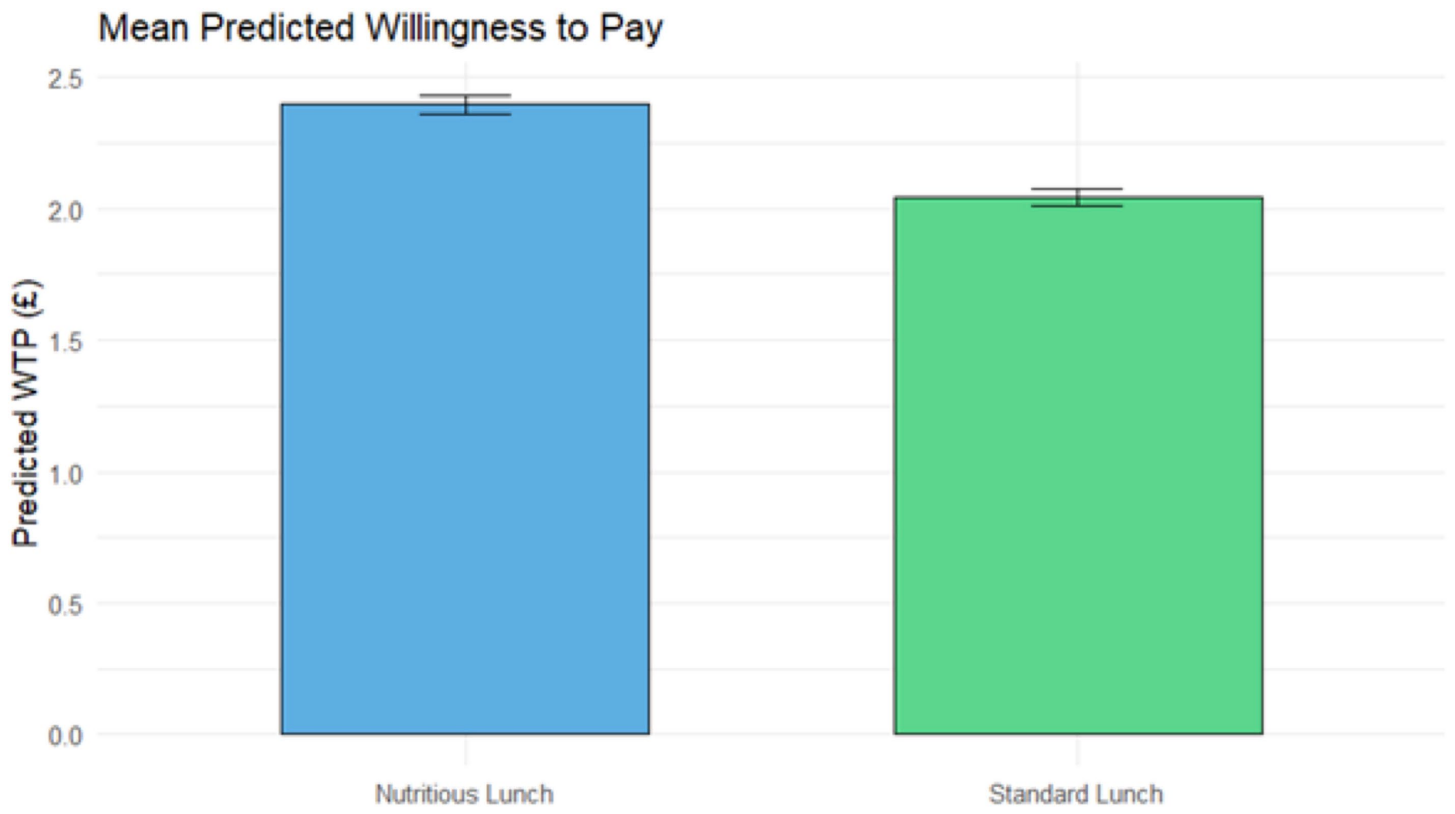
Mean predicted willingness to pay for standard and nutritious school lunches with 95% confidence intervals.

Figure 5 presents the predicted WTP for both a standard and a nutritious school lunch, derived from the fitted regression models. The average predicted WTP for a standard lunch was £2.05 (median = £1.99), while for a nutritious lunch it was £2.40 (median = £2.35). The average predicted WTP was £0.35 higher for a nutritious lunch than for a standard one.

The findings were consistent with Hypothesis 1, with parents reporting higher WTP for a nutritious school lunch than for a standard school lunch (Table 3). Hypothesis 2 was also supported, as gross household income was positively associated with WTP. Consistent with Hypothesis 3, parents whose children rarely or inconsistently consumed school lunches, or who reported less favorable perceptions of current school meals, had lower WTP values.

### 3.7 Sensitivity analysis

The Tobit models yielded coefficients similar in magnitude and direction to the OLS estimates, though slightly attenuated, suggesting minimal impact of bounded responses on the primary results. Gender and mild food insecurity were excluded from the final Tobit model for WTP for a standard school lunch due to perfect collinearity with other predictors and interaction terms. The Tobit results are reported in Supplementary Table S5.

## 4 Discussion

### 4.1 Summary of findings

This study is, to our knowledge, the first UK analysis to use contingent valuation to estimate parental WTP for subsidised school meals. The findings indicate that most parents (88.4%) who are currently ineligible for FSMs are WTP toward the cost of a school lunch, with a mean predicted WTP of £2.05 for a standard lunch and £2.40 for a nutritious lunch. These values fall below the current average cost of provision (£2.70), confirming that a co-payment model would require continued government subsidy, but at a substantially reduced level compared with full universal free provision. The £0.35 premium for nutritious meals demonstrates that parents value quality and are willing to invest more when nutritional standards are met, a finding with direct implications for school food standards (33, 34).

#### 4.1.1 Income and willingness to pay

A notable finding was the divergent role of income across the two model components. Among those WTP, higher income was associated with higher WTP amounts, consistent with standard economic theory and international evidence (35, 36). However, the logistic model revealed that the likelihood of being unwilling to pay also increased with income. This finding warrants further investigation, as the present data do not capture the reasons behind non-participation.

#### 4.1.2 Food insecurity as a barrier to participation

Very severe food insecurity in the past month was a strong barrier to being WTP, highlighting that the households most likely to benefit nutritionally from subsidised school meals are also the least able to contribute financially. This finding highlights the importance of retaining full exemptions for food insecure households within any co-payment scheme and has direct public health implications: without targeted support, a co-payment model could inadvertently exclude the children at greatest nutritional risk. The association between moderate 12-month food insecurity and lower WTP amounts further reinforces that financial hardship constrains both the decision to participate and the capacity to contribute.

#### 4.1.3 Breakfast club attendance

The finding that breakfast club attendance was associated with approximately three times higher odds of being unwilling to pay for school lunches has not been previously reported. This association may reflect cumulative financial pressures among households already contributing to breakfast provision, though the underlying mechanisms could not be assessed in this study (37, 38). This finding has practical implications for integrated school food policy such as improving menu transparency, increasing parental engagement with school food provision, and offering flexible participation options (e.g., part-week meal uptake). These approaches may complement bundled meal packages in improving uptake.

#### 4.1.4 Gender differences in WTP

Female respondents consistently reported lower WTP than male respondents for both standard and nutritious school lunches. Gender differences in WTP have been reported in other valuation studies, with women typically reporting lower absolute WTP values, potentially reflecting income constraints or differences in household budgeting responsibilities (39). Interpretation in this context, however, warrants caution. Although household food purchasing and provisioning responsibilities are increasingly shared, evidence from UK time-use studies indicates that women continue to undertake a disproportionate share of unpaid household and food-related labour (40). This may be associated with lower stated WTP, though the finding may reflect gendered differences in financial autonomy or spending priorities within households.

#### 4.1.5 Engagement with school food and the role of perceived quality

Consistent with Hypothesis 3, parents whose children regularly consumed school lunches and who held positive perceptions of school food quality were WTP more, while disengagement, exercised through reliance on packed lunches or negative quality perceptions was associated with lower WTP. These findings align with evidence from other studies (36, 41) and suggest that perceived quality and existing engagement with school food are factors associated with participation. Policies that improve transparency such as involving parents in menu planning, communicating nutritional standards, and publicising compliance with school food regulations may increase both perceived quality and WTP (42).

### 4.2 Comparison with international evidence

Our findings are broadly consistent with the international literature on parental WTP for school meals. In Canada, over 90% of parents were willing to participate in a universal school food programme, with a mean WTP of approximately CAD 4.68 (£2.92 at 2022 exchange rates), and income was a strong positive predictor (35). In Indonesia, around 30% of parents were WTP above baseline cost, with income and positive attitudes as key predictors (36). Similar patterns were reported in Estonia (41). While the magnitude of WTP and the strength of specific predictors vary by setting, the consistent role of income, quality perceptions, and engagement across countries reinforces the generalisability of the core finding: WTP toward school meals is shaped by both economic capacity and perceived value.

### 4.3 Strengths and limitations

This study has several strengths. It is the first UK study to apply contingent valuation to estimate WTP for a subsidised school meal, providing novel evidence to inform an active policy debate. The two-stage modeling approach captures both the decision to participate and the intensity of WTP, providing a more complete picture than studies that examine only one dimension. The inclusion of all theoretically relevant covariates (a “maximum model” approach) reduces the risk of omitted variable bias. The relatively large, geographically diverse sample of 1,009 parents enhances generalisability.

Several limitations should be acknowledged. First, contingent valuation is subject to hypothetical bias: stated WTP may exceed what respondents would actually pay in a real-world scenario (43, 44). Our estimates should therefore be interpreted as upper-bound valuations. Second, the provision of an anchor (£2.70 average cost) and the £5 upper bound may have influenced responses through anchoring effects and range truncation, respectively (45, 46). Third, the two WTP questions were asked in a fixed order (standard lunch first), which may have inflated the WTP for the nutritious lunch through a contrast or yea-saying effect. Fourth, the nutritious school lunch scenario did not provide respondents with a specific definition of nutritional quality. Perceptions of what constitutes a ‘nutritious’ meal are likely to vary by socioeconomic background, cultural context, and nutritional knowledge, meaning the observed WTP premium may reflect heterogeneous interpretations of the quality improvement rather than a uniform valuation. Fifth, Food insecurity was self-reported and may be subject to underreporting due to social desirability or stigma, potentially underestimating the true prevalence and strength of its association with WTP.

Sixth, ethnicity was coded as a binary variable (White vs. ethnic minority). This aggregation obscures substantial heterogeneity between ethnic groups, future studies should examine WTP patterns across disaggregated ethnic categories. Finally, the cross-sectional design precludes causal inference, and the reliance on self-reported data introduces potential social desirability and recall biases.

### 4.4 Policy implications

The findings from this study have several implications for public health policy. First, the high proportion of parents willing to contribute (88.4%) suggests that a subsidised co-payment model is acceptable in principle to most UK families currently ineligible for FSMs. If designed appropriately, such a scheme could substantially expand children’s access to school meals, which are associated with improved dietary quality, reduced childhood overweight, better educational attainment and financial savings for households, while reducing the fiscal burden on government relative to full universal provision (36, 47).

Second, the consistent finding that food insecure households face the greatest barriers to participation reinforces the need for a progressive policy design in which the most vulnerable families are fully exempt from contributions. Without such protections, a co-payment model risks widening rather than narrowing dietary and health inequalities.

Third, the strong association between perceived meal quality and WTP suggests that investment in school food standards and in communicating those standards to parents could simultaneously improve dietary outcomes and increase programme sustainability by raising household contributions. There is also a need for integrated school food strategies that consider the full school day, rather than treating individual meal programmes in isolation, i.e., breakfast clubs and school lunch. Bundled meal policies and transparent pricing may reduce financial barriers and improve overall uptake.

Lastly, if the government were to set a co-payment at £2.00, the demand curve suggests that approximately 55% of currently ineligible families would participate for a standard lunch, rising to approximately 75% if nutritional quality were assured. At this price point, the government subsidy required would be approximately £0.70 per meal per participating child, substantially less than the full £2.70 required under universal free provision. For the 30–45% of families unwilling or unable to contribute at the £2.00 level, targeted subsidies or full exemptions would be needed, particularly for food insecure households. Eligibility verification mechanisms may be required to ensure equitable targeting of exemptions. These trade-offs between participation rates, subsidy levels, and equity highlight the importance of setting the co-payment at a level that balances fiscal sustainability with broad access to this public health intervention.

### 4.5 Future research

Future research should evaluate the behavioral sustainability of co-payment models under real-world conditions through experimental or quasi-experimental designs testing different price points, subsidy levels, and quality assurance mechanisms. Longitudinal studies are needed to understand how WTP evolves in response to economic conditions, inflation, and changing social norms. Discrete choice experiments could complement contingent valuation by capturing the relative importance of specific meal attributes (e.g., organic sourcing, menu variety) in driving parental preferences. Such evidence would be invaluable for developing scalable, inclusive, and financially sustainable school food policies. Additionally, future contingent valuation studies in this area should incorporate follow-up questions for respondents expressing zero WTP, to distinguish genuine unwillingness from protest responses, a well-documented phenomenon in stated-preference research (27). For example, respondents could be asked to select a reason for non-participation from a predefined list, such as ‘I believe school meals should be free’, ‘I prefer to provide packed lunches’, ‘I cannot afford to contribute’, or ‘I do not value school meals’ enabling researchers to separate principled objections from true zero valuations, and to produce adjusted WTP estimates that exclude protest responses.

## 5 Conclusion

This study demonstrates that 88.4% of UK parents of primary school children who are currently ineligible for free school meals are willing to contribute toward the cost of a subsidised school lunch, and that this willingness increases when nutritional quality is assured. These findings provide actionable evidence for policymakers seeking to expand school meal provision. Specifically, a co-payment model, set at a price point around £1.50–£2.00, combined with eligibility-based exemptions for financially vulnerable and food insecure households could achieve high levels of participation while substantially reducing the government subsidy required per meal. This also requires complementary strategies to improve perceived quality and build parental trust.

## Data Availability

The authors confirm that the data supporting the findings of this study are available within the article and its supplementary materials. The underlying individual-level survey data were collected under licence from YouGov and are not publicly available due to commercial restrictions. Aggregated results, analysis code (R scripts), and the full survey instrument are provided in the supplementary materials.

## The group member of on behalf of the mandala consortium

**Other members of consortium (not listed as authors) include: Jean Adams, Thomas Burgoine, Noah Cooke, Oliver Francis, Kelly Parsons, Catrin Pedder Jones, Alexia Sawyer, Roxanne Armstrong-Moore, Struan Tait, Jaideep Padbhu, Jagjit Srai, Soujanya Mantravadi, Ettore Settanni, Thomas Ball, Andrew Balmford, Andrew Bladon, Lynn Dicks, University of Cambridge; Steve Cummins, Robert Greener, Sophie Hedges, London School of Hygiene and Tropical Medicine; Kehinde Adeosun, Isabel Nuñez Salazar, University of Liverpool; Owen Nicholas, University College London; Thijs van Rens, University of Warwick; Professor Richard Smith, University of Exeter; Anna Taylor, Shona Goudie, The Food Foundation; Leticija Petrovic, The Food Foundation and Birmingham City Council.
