# Supplementary material for: Financial accessibility of school meals: evidence from parental willingness to co-pay in the UK
Source: Front Public Health. 2026 Jun 11;14:1839749. doi: 10.3389/fpubh.2026.1839749 (PMC13294095; doi:10.3389/fpubh.2026.1839749)
Supplement: Supplementary file 1 [file Table_1.docx]

**Supplementary File**

**Supplementary file 1: Full Survey Instrument**

For pupils who are currently not eligible for a FSM, instead of asking households to pay the full cost for a school meal, we explore a subsidised alternative whereby households part-pay, and the government subsidise the remaining cost. Only households who are currently not eligible for a FSM would be given the opportunity to contribute. Under this hypothetical scenario, to understand how much households would be willing to contribute towards a school lunch, we designed a survey using the contingent valuation approach. The overall aim was to estimate the average household willingness to contribute for a primary school lunch.

The survey instrument was designed for self-completion and administered by The Food Foundation through YouGov, a global market survey provider that conducts online public opinion surveys on a variety of topics. After the survey was first developed, it was piloted and revised with the study team and a public advisory group (based at University of Birmingham). The start of the survey contained a description of the current policy for school lunches in England, and an outline of how a co-payment policy would be designed. Each household was then asked if they would opt for a school lunch and then asked to indicate a maximum willingness to contribute towards a school lunch.

Questions used in the survey:

Q1: Are you willing to contribute towards the cost of a school lunch for your own child if you pay some of the cost and the government pays the rest?

- Yes

- No

Q1b: If you aren’t willing to contribute towards the cost of a school lunch, why is that?

Q2: Please state the maximum amount you would be willing to contribute (per day) towards the cost of a school lunch. Please assume there would be no change to the quality of a school lunch. As a guide, the average cost of a school lunch in England is £2.70 but the amount you say could be higher or lower than this amount, it is up to you.

Please type in £ and pence (0.00) value between 0.01 and 5.00.

Q3: Now please select the maximum amount you would be willing to contribute (per day) towards the cost of a school lunch assuming the food is nutritious and high quality.

Please type in £ and pence (0.00) value between 0.01 and 5.00.

Please can you explain your reasons for your answer to the previous two questions, please tick all that apply.

- This is how much I value a subsidised school lunch.

- I prefer not to contribute towards the cost of school lunches.

- I cannot afford to pay more.

- I would rather make a packed lunch for my child(ren)

Other (please specify)

Q5: If your child were to receive a subsidised school lunch every day, how frequently would you prefer to pay?

Options given: Daily, Weekly, Monthly, At start of each half term, At start of each term, At start of each academic year (yearly), I am not able to pay, I do not want to pay.

Q6: How many adults (those age over 16) live in your household?

Choose from:1,2,3,4 or more than 4.

Q7: The options below show various possible sources of income. Can you please tell us which of these your household receives? Please select all boxes that apply.

- Earnings from employment or self-employment

- State retirement pension

- Personal pension or pension from formal employer

- Job-seekers allowance

- Employment and support allowance

- Income support

- Pension credit

- Working tax credit.

- Child tax credit

- Child benefit

- Housing benefit

- Universal credit

- Disability benefit (Personal independence plan/Disability living allowance)

- Other state benefits

- No source of income

Q8: Gross HOUSEHOLD income is the combined income of all those earners in a household from all sources, including wages, salaries, or rents and before tax deductions. What is your gross household income?

- under £5,000 per year

- £5,000 to £9,999 per year

- £10,000 to £14,999 per year

- £15,000 to £19,999 per year

- £20,000 to £24,999 per year

- £25,000 to £29,999 per year

- £30,000 to £34,999 per year

- £35,000 to £39,999 per year

- £40,000 to £44,999 per year

- £45,000 to £49,999 per year

- £50,000 to £59,999 per year

- £60,000 to £69,999 per year

- £70,000 to £99,999 per year

- £100,000 to £149,999 per year

- £150,000 and over

- Don't know.

- Prefer not to answer.

Q9: Thinking about the last month, have you? Please tick all that apply.

- Had smaller meals than usual or skip meals because you couldn’t afford or get access to food.

- Ever been hungry but not eaten because you couldn’t afford or get access to food.

- Not eaten for a whole day because you couldn’t afford or get access to food.

- None of these

Q10: Thinking about the last 12 months, have you? Please tick all that apply.

- Had smaller meals than usual or skip meals because you couldn’t afford or get access to food.

- Ever been hungry but not eaten because you couldn’t afford or get access to food.

- Not eaten for a whole day because you couldn’t afford or get access to food.

- None of these.

Q11: Does your child(ren) attend a school breakfast club?

- Yes

- No

- 99. Not applicable, school does not offer breakfast club.

Q11b: Do you pay for the breakfast club?

- Yes

- No, the school provides the club free of charge.

Q11c: How much do you pay for the breakfast club? Please type in £ and pence (0.00) value between 0.01 and 8.00.

Q11d: How often do you pay for the breakfast club?

Choose from: Daily, Weekly, Monthly, At start of each half term, At start of each term, At start of each academic year (yearly), Not sure

Q12: Does your child/children typically eat lunch provided by the school or bring a packed lunch from home?

- Yes, they eat lunch provided by the school every day.

- No, they bring a packed lunch from home every day.

- Mixed, combination of school lunches and packed lunches

Q13: Can you provide an estimate on how much you spend on a typical packed lunch.

Please type in £ and pence (0.00) value between 0.01 and 10.00.

Q14: Are you familiar with the lunch menu offered by your child's school?

- Yes

- No

Q15: How would you rate the choice and variety of food available during a school lunch:

- High (Lots of choice and variety)

- Medium (Average choice and variety)

- Low (Not much choice and variety)

- 99. Not sure

Q16: Please rate how you would value the following benefits from your child receiving a paid for school lunch.

For each benefit listed below, they asked to rate from 1 (very important) to 3 (this is not important to me). Were also given a ‘not sure’ option.

- Health and nutrition

- Improved social relationships

- Less food waste

- Enhanced support for the school's food services, potentially leading to increased job opportunities within the school catering department

- Improved educational attainment

- Opportunity to try new foods

**Supplementary File 2- WTP Variables**

Table S1: Co-variates hypothesised to influence parent’s WTP for government subsidised school meal program.

| Variables | Explanation | Justification | Source |
| --- | --- | --- | --- |
| Gender | Parent/guardian’s gender. | Males/females have different dietary behaviours that may impact WTP | Developed by the research team |
| Age | Parent/guardian’s age in years. | Younger and older parents may have different priorities and spending capacities and therefore may value convenience or nutrition differently which will influence WTP |  |
| Household size (adults) | Number of adults aged > 16 years living in the household. | Larger households potentially have a higher disposable income which could affect ability to pay for school lunches. |  |
| Ethnicity | Self-reported ethnic group of parent/guardian. | Having ethnic minority status may affect WTP. |  |
| Gross Household Annual Income | Household income measure derived from survey responses. | Substantial economic evidence shows that ability to pay directly affects WTP. |  |
| Working | Parent/guardian’s employment status. | Being employed may affect WTP |  |
| Food insecurity Index in the last month | Self-reported levels of food insecurity experienced in the last month | A higher level of food insecurity implies a lower ability to pay and therefore lower WTP. Previous studies show severe food insecurity as a factor reducing WTP for school feeding programs. |  |
| Food insecurity Index in the last 12 months | Self-reported levels of food insecurity experienced in the last 12 months | As above. |  |
| Attends school breakfast club | Children currently attend school breakfast club | Parents whose children attend breakfast clubs are likely more engaged with school meal programs in general. This engagement suggests a reliance or trust in the school's food services, increasing their WTP. |  |
| Pays for breakfast club | Parents/guardians pay for breakfast club | Already paying for breakfast club may impact additional WTP for school lunches. |  |
| Typically eat school lunch. | Children have a school lunch | Parents whose children typically have a school lunch have demonstrated that they value the service enough to use it regularly. This habitual behaviour indicates a higher perceived value, thus a higher WTP. |  |
| Perception of current offer | Self-reported perception of school lunch quality | Demand theory predicts that the more satisfaction gained from a ‘good’ the higher the WTP |  |
| Familiarity with school lunch. | Self-reported parents/guardians’ familiarity with the school lunch provided | The more familiar parents are with the school lunch offerings, the more informed they are about the quality and benefits, which can lead to a higher WTP. |  |

**Supplementary File 3- Histogram of WTP variables**

Figure S1: Histogram of parents’ willingness to pay (WTP) for a standard school lunch (£). The red dashed line indicates the mean WTP (£2.03).


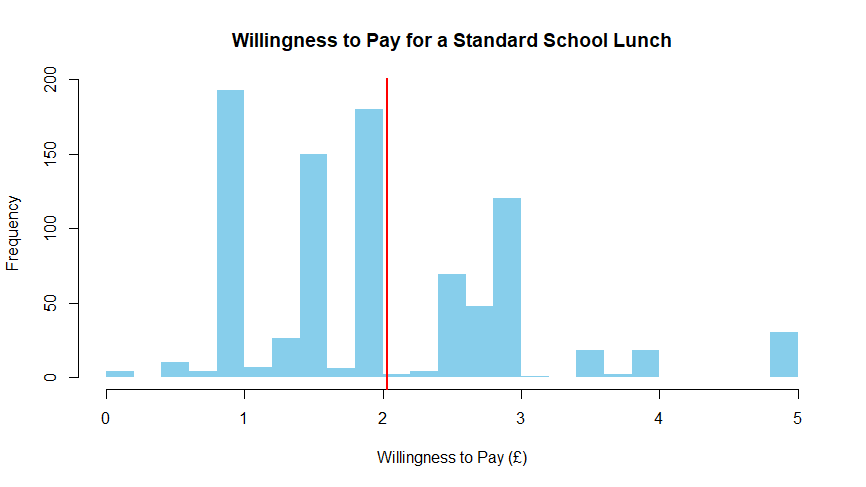


Figure S2: Histogram of parents’ willingness to pay (WTP) for a Nutritious school lunch (£). The red dashed line indicates the mean WTP (£2.37).


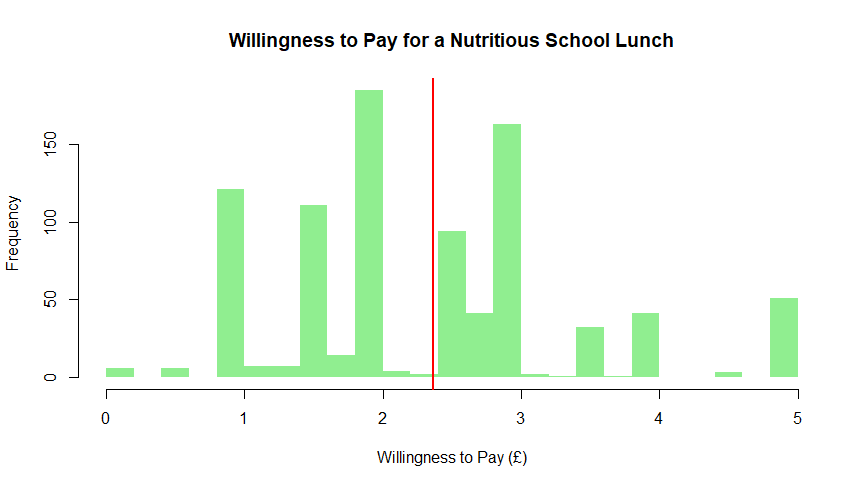


**Supplementary File 4: Formal Model Specification and Model Diagnostics**

### Main Econometric Analysis

#### Logistic Regression Model

The decision to WTP was modelled using a fixed-effects logistic regression:

$\text{logit}(P_{i})=ln(\frac{P_{i}}{1-P_{i}})=\beta_{0}+\beta_{1}X_{i}+\varepsilon_{i}$ *Equation 2*

where $P_{i}$ is the probability that household $i$ is not willing to contribute (NotWTP = 1).

#### Linear Regression Model

The OLS specification was defined as:

$WTP_{i}=\beta_{0}+\beta_{1}X_{i}+\varepsilon_{i}$ Equation 3

where $WTP_{i}$ represents the continuous amount (in £) that household $i$ is WTP.

**Model diagnostics**

Both OLS models met key regression assumptions. The distributions were slightly right-skewed (skewness = 0.97, kurtosis = 1.01) and (skewness = 0.66, kurtosis = 0.23) for the nutritious school lunch respectively, but was considered sufficiently symmetric for linear modelling(1,2). Residual plots confirmed approximate linearity and constant variance, Durbin–Watson statistics (1.98 and 2.11) indicated no autocorrelation, and variance inflation factors (VIF < 2) confirmed the absence of multicollinearity. Mild heteroskedasticity was corrected using HC1 robust standard errors(3–5). Residual non-normality at the tails was judged negligible given the sample size. The models explained approximately 18% of variance in WTP (R² = 0.184 and 0.185 for the standard and nutritious lunch models, respectively). While modest, this level of explanatory power is consistent with comparable WTP survey studies and suggests that unmeasured factors, such as personal dietary beliefs, trust in schools, peer influence, or convenience preferences may also shape parental valuations.

**Supplementary 5- Logistic regression: Alternative model specifications**

*Table S2: Factors associated with not being willing to contribute towards the cost of a school lunch*

| **Barriers to Parents’ Willingness to Co-Pay for School Lunches** | | | |
| --- | --- | --- | --- |
| **Dependent variable: Unwilling to Co-Pay (1 = No, 0 = Yes)** | | | |
| **Variable** | **Model 1** | **Model 2** | **Model 3** |
| Female | 0.32 (0.30) | 0.30*** (0.31) | 0.27** (0.41) |
| Age (years) | 0.87*** (0.02) | 0.88*** (0.02) | 0.90*** (0.03) |
| Income | 1.23*** (0.04) | 1.22*** (0.04) | 1.12* (0.06) |
| Adult household size | 0.91 (0.29) | 0.86 (0.30) | 0.96 (0.36) |
| Moderate food insecurity (last month) |  | 1.82 (1.07) | 0.93 (1.49) |
| Severe food insecurity (last month) |  | 9.09 (1.65) | 11.91 (1.75) |
| Very severe food insecurity (last month) |  | 48.12** (1.33) | 55.26** (1.52) |
| Mild food insecurity (12-month) |  | <0.001 (801.81) | <0.001 (843.44) |
| Moderate food insecurity (12-month) |  | 0.42 (0.85) | 0.73 (1.00) |
| Severe food insecurity (12-month) |  | 0.27 (1.29) | 1.13 (1.62) |
| Very severe food insecurity (12-month) |  | <0.001 (4,595.77) | <0.001 (4,588.59) |
| Perception of school lunch (Medium) |  |  | 0.44 (0.44) |
| Perception of school lunch (Low) |  |  | 0.86 (0.55) |
| Typically eat lunch (No) |  |  | 1.60 (0.50) |
| Typically eat lunch (Mixed) |  |  | 1.26 (0.44) |
| Attends school breakfast club |  |  | 2.67** (0.37) |
| Working |  |  | 2.46 (1.02) |
| Ethnicity (Ethnic minority) |  |  | 0.97 (0.59) |
| Female × Income |  |  |  |
| Constant | 6.66*** (0.97) | 7.36*** (1.03) | 1.19 (1.69) |
|  | | | |
| **Observations** | 856 | 856 | 652 |
| **Log Likelihood** | –221.26 | –213.25 | –122.22 |
| **Akaike Inf. Crit.** | 452.53 | 450.50 | 282.44 |

*Note: ***p < 0.001; **p < 0.01;* ***^*^****p < 0.05; p < 0.10.* Robust (HC1) standard errors in parentheses for all three models.

**Supplementary 6- Linear regression: Alternative model specifications**

Table S3: Alternative Model Specifications and Statistics for a Standard School Lunch.

| **Dependent variable: WTP (£) for a Standard School Lunch** | | | | |
| --- | --- | --- | --- | --- |
| **Variable** | **Model 1** | **Model 2** | **Model 3** | **Model 4** |
| Female | –0.27*** (0.07) | –0.23** (0.07) | –0.24*** (0.08) | –0.26*** (0.08) |
| Age (years) | –0.02*** (0.01) | –0.02*** (0.01) | –0.02*** (0.01) | –0.02*** (0.01) |
| Income | –0.56 (0.41) | 0.05*** (0.01) | 0.04*** (0.01) | 0.04*** (0.01) |
| Adult household size | –0.08 (0.07) | –0.07 (0.07) | –0.05 (0.07) | –0.02 (0.08) |
| Moderate food insecurity (last month) | –0.35*** (0.10) | 0.17 (0.23) | 0.06 (0.24) | 0.08 (0.24) |
| Severe food insecurity (last month) | –0.68** (0.28) | –0.08 (0.37) | –0.13 (0.40) | –0.13 (0.40) |
| Very severe food insecurity (last month) | –0.44 (0.49) | 0.10 (0.49) | 0.03 (0.48) | 0.12 (0.49) |
| Mild food insecurity (12 months) |  | –0.53** (0.27) | –0.29 (0.28) | –0.31 (0.28) |
| Moderate food insecurity (12 months) |  | –0.50*** (0.16) | –0.42** (0.16) | –0.43*** (0.16) |
| Severe food insecurity (12 months) |  | –0.42 (0.26) | –0.32 (0.27) | –0.28 (0.27) |
| Very severe food insecurity (12 months) |  | –0.37 (0.70) | –0.21 (0.68) | –0.29 (0.68) |
| Attends school breakfast club |  |  | 0.07 (0.09) | 0.05 (0.09) |
| Typically eat lunch (No) |  |  | –0.29*** (0.09) | –0.33*** (0.09) |
| Typically eat lunch (Mixed) |  |  | –0.19* (0.09) | –0.20* (0.09) |
| Perception of school lunch (Medium) |  |  | –0.32*** (0.11) | –0.32*** (0.11) |
| Perception of school lunch (Low) |  |  | –0.49*** (0.13) | –0.51*** (0.13) |
| Working |  |  |  | 0.10 (0.13) |
| Ethnicity (Ethnic minority) |  |  |  | –0.21* (0.13) |
| Constant | 3.12*** (0.26) | 2.83*** (0.27) | 3.14*** (0.30) | 3.14*** (0.33) |
|  | | | | |
| **Observations** | 756 | 756 | 622 | 609 |
| **R²** | 0.06 | 0.10 | 0.15 | 0.16 |
| **Adjusted R²** | 0.05 | 0.09 | 0.12 | 0.13 |
| **AIC** | 2060.75 | 1641.05 | 1611.10 | 1600.94 |

*Note: ***p < 0.001; **p < 0.01; *p < 0.05. Robust (HC1) standard errors in parentheses.*

Table S4: Alternative Model Specifications and Statistics for the WTP for a Nutritious School Lunch

| **Dependent variable: WTP (£) for a Nutritious School Lunch** | | | | |
| --- | --- | --- | --- | --- |
| **Variable** | **Model 1** | **Model 2** | **Model 3** | **Model 4** |
| Female | –0.19** (0.08) | –0.19** (0.08) | –0.17** (0.08) | –0.17** (0.08) |
| Age (years) | –0.02*** (0.01) | –0.02*** (0.01) | –0.01* (0.01) | –0.01* (0.01) |
| Income | 0.08*** (0.01) | 0.07*** (0.01) | 0.06*** (0.01) | 0.06*** (0.01) |
| Adult household size | –0.08 (0.07) | –0.08 (0.07) | –0.06 (0.07) | –0.06 (0.08) |
| Moderate food insecurity (last month) | –0.23** (0.11) | 0.29 (0.25) | 0.30 (0.24) | 0.21 (0.26) |
| Severe food insecurity (last month) | –0.60** (0.30) | –0.05 (0.40) | 0.10 (0.40) | –0.22 (0.43) |
| Very severe food insecurity (last month) | –0.49 (0.51) | –0.02 (0.53) | –0.02 (0.53) | –0.06 (0.52) |
| Mild food insecurity (12 months) |  | –0.62** (0.29) | –0.64** (0.28) | –0.45 (0.30) |
| Moderate food insecurity (12 months) |  | –0.52*** (0.17) | –0.58*** (0.16) | –0.52*** (0.18) |
| Severe food insecurity (12 months) |  | –0.58** (0.28) | –0.68** (0.27) | –0.61** (0.29) |
| Very severe food insecurity (12 months) |  | –0.57 (0.76) | –0.67 (0.74) | –0.49 (0.74) |
| Attends school breakfast club |  |  | 0.12 (0.09) | 0.16 (0.10) |
| Typically eat lunch (No) |  |  | –0.43*** (0.09) | –0.42*** (0.10) |
| Typically eat lunch (Mixed) |  |  | –0.37*** (0.09) | –0.34*** (0.10) |
| Perception of school lunch (Medium) |  |  |  | –0.13 (0.12) |
| Perception of school lunch (Low) |  |  |  | –0.25* (0.14) |
| Constant | 2.83*** (0.29) | 2.92*** (0.29) | 3.05*** (0.29) | 3.05*** (0.33) |
|  | | | | |
| **Observations** | 756 | 756 | 728 | 622 |
| **R²** | 0.11 | 0.12 | 0.15 | 0.18 |
| **Adjusted R²** | 0.10 | 0.11 | 0.14 | 0.15 |
| **AIC** | 2174.98 | 2172.41 | 2059.57 | 1744.28 |

*Note: ***p < 0.001; **p < 0.01; *p < 0.05. Robust (HC1) standard errors in parentheses.*

**Supplementary File 7- Sensitivity Analysis**

**Table S5- Tobit Regression for Parent’s WTP for a Standard and Nutritious School Lunch**

| **Dependent variable: WTP (£) for a school lunch (Tobit regression)** | | |
| --- | --- | --- |
| **Variable** | **Standard Lunch** | **Nutritious Lunch** |
| (Intercept) | 2.05*** (0.40) | 2.98*** (0.35) |
| Income | 0.19*** (0.04) | 0.05*** (0.01) |
| Age (years) | –0.02** (0.01) | –0.01 (0.01) |
| Female | – | –0.21* (0.08) |
| Ethnicity (Ethnic minority) | –0.16 (0.12) | –0.34* (0.13) |
| Adult household size | 0.40** (0.14) | –0.01 (0.08) |
| Moderate food insecurity (last month) | 0.13 (0.23) | 0.24 (0.25) |
| Severe food insecurity (last month) | –0.05 (0.39) | –0.21 (0.42) |
| Very severe food insecurity (last month) | 0.19 (0.48) | 0.08 (0.52) |
| Mild food insecurity (12 months) | –0.40 (0.27) | –0.49 (0.30) |
| Moderate food insecurity (12 months) | –0.42** (0.16) | –0.55** (0.18) |
| Severe food insecurity (12 months) | –0.35 (0.26) | –0.59* (0.29) |
| Very severe food insecurity (12 months) | –0.21 (0.66) | –0.62 (0.72) |
| Perception of school lunch (Medium) | –0.42** (0.15) | –0.13 (0.12) |
| Perception of school lunch (Low) | –0.76*** (0.20) | –0.26 (0.14) |
| Typically eats school lunch (No) | –0.18 (0.27) | –0.48*** (0.10) |
| Typically eats school lunch (Mixed) | –0.61** (0.24) | –0.38*** (0.10) |
| Attends school breakfast club | 0.06 (0.09) | 0.13 (0.10) |
| Working | 0.19 (0.13) | 0.15 (0.14) |
| Income × Adult household size | –0.07*** (0.02) | – |
| Perception (Low) × Typically eat lunch (Mixed) | 0.75* (0.31) | – |
|  | | |
| **AIC** | 1601.47 | 1708.60 |

*Note: ***p < 0.001; **p < 0.01; *p < 0.05. ‘–’ indicates variable not included in the model.*

**Supplementary File 8 – R Do File**
library(foreign)

WTPdataR <- read.csv(file.choose(), header = TRUE)

library(ggplot2)

library(lattice)

str(WTPdataR)

# Create a numeric age variable from the range

WTPdataR$Age_continuous_num <- as.numeric(

sapply(WTPdataR$Age_continuous, function(x) {

if (grepl("-", x)) {

# Split the range and take the average

parts <- as.numeric(unlist(strsplit(x, "-")))

mean(parts)

} else if (grepl("Under", x, ignore.case = TRUE)) {

20 # or another midpoint for "Under 25" etc.

} else if (grepl("65", x, ignore.case = TRUE)) {

70 # approximate for "65+" or similar

} else {

NA

}

})

)

library(stringr)

install.packages("stringr")

library(stringr)

# Convert income ranges to numeric midpoints (in units of £10,000)

WTPdataR$Income_continuous_num <- sapply(WTPdataR$Income_continuous, function(x) {

x <- trimws(x)

if (grepl("to", x)) {

nums <- as.numeric(str_extract_all(x, "[0-9]+", simplify = TRUE))

mean(nums) / 10000

} else if (grepl("Under", x, ignore.case = TRUE)) {

1 # £10,000 represented as 1 unit of 10,000

} else if (grepl("Don't know|Prefer not", x, ignore.case = TRUE)) {

NA

} else if (grepl("[0-9]", x)) {

as.numeric(gsub(",", "", str_extract(x, "[0-9]+"))) / 10000

} else {

NA

}

})

class(WTPdataR)

Q

WTPdataR$AdultHHsize_3cat <- ifelse(WTPdataR$AdultHHsize_categorical == 1, 1,

ifelse(WTPdataR$AdultHHsize_categorical == 2, 2, 3))

table(WTPdataR$AdultHHsize_categorical, WTPdataR$AdultHHsize_3cat)

summary(WTPdataR$AdultHHsize_3cat)

WTPdataR$Perceptionofschlunch_3cat <- ifelse(WTPdataR$Perceptionofschlunch == 1, 1,

ifelse(WTPdataR$Perceptionofschlunch == 2, 2, 3))

WTPdataR$Perceptionofschlunch_3cat <- factor(

WTPdataR$Perceptionofschlunch_3cat,

levels = c(1, 2, 3),

labels = c("High", "Medium", "Low/Not sure")

)

WTPdataR$Attendschbreakfastclub[WTPdataR$Attendschbreakfastclub == 99] <- NA

table(WTPdataR$Attendschbreakfastclub, useNA = "ifany")

WTPdataR$Attendschbreakfastclub <- factor(

WTPdataR$Attendschbreakfastclub,

levels = c(0, 1),

labels = c("No", "Yes")

)

WTPdataR$Attendschbreakfastclub <- as.numeric(as.character(WTPdataR$Attendschbreakfastclub))

table(WTPdataR$Attendschbreakfastclub, useNA = "ifany")

names(WTPdataR)

original <- read.csv(file.choose(), header = TRUE)

WTPdataR$Attendschbreakfastclub <- original$Attendschbreakfastclub

table(WTPdataR$Attendschbreakfastclub, useNA = "ifany")

WTPdataR$Attendschbreakfastclub[WTPdataR$Attendschbreakfastclub == 99] <- NA

table(WTPdataR$Attendschbreakfastclub, useNA = "ifany")

WTPdataR$AdultHHsize_3cat <- ifelse(WTPdataR$AdultHHsize_3cat == "1 adult", 1,

ifelse(WTPdataR$AdultHHsize_3cat == "2 adults", 2,

ifelse(WTPdataR$AdultHHsize_3cat == "3+ adults", 3, NA)))

WTPdataR$Perceptionofschlunch_3cat <- ifelse(WTPdataR$Perceptionofschlunch_3cat == "High", 1,

ifelse(WTPdataR$Perceptionofschlunch_3cat == "Medium", 2,

ifelse(WTPdataR$Perceptionofschlunch_3cat == "Low/Not sure", 3, NA)))

WTPdataR$Perceptionofschlunch_3cat <- ifelse(WTPdataR$Perceptionofschlunch_3cat == "High", 1,

ifelse(WTPdataR$Perceptionofschlunch_3cat == "Medium", 2,

ifelse(WTPdataR$Perceptionofschlunch_3cat == "Low/Not sure", 3, NA)))

table(WTPdataR$Perceptionofschlunch_3cat, useNA = "ifany")

WTPdataR$Freqofpayingbreakfast_4cat <- ifelse(WTPdataR$Freqofpayingbreakfast == 1, 1,

ifelse(WTPdataR$Freqofpayingbreakfast == 2, 2,

ifelse(WTPdataR$Freqofpayingbreakfast == 3, 3,

ifelse(WTPdataR$Freqofpayingbreakfast %in% c(4, 5, 6), 4,

ifelse(WTPdataR$Freqofpayingbreakfast == 99, NA, NA)))))

table(WTPdataR$Freqofpayingbreakfast, WTPdataR$Freqofpayingbreakfast_4cat, useNA = "ifany")

summary(WTPdataR$Freqofpayingbreakfast_4cat)

WTPdataR$Income_categorical_6cat <- ifelse(WTPdataR$Income_categorical == 1, 1, # under £5,000

ifelse(WTPdataR$Income_categorical %in% 2:6, 2, # £5,000–£29,999

ifelse(WTPdataR$Income_categorical %in% 7:12, 3, # £30,000–£69,999

ifelse(WTPdataR$Income_categorical %in% 13:14, 4, # £70,000–£149,999

ifelse(WTPdataR$Income_categorical == 15, 5, # £150,000+

ifelse(WTPdataR$Income_categorical %in% c(16,17), NA, NA)))))) # Don't know / Prefer not

table(WTPdataR$Income_categorical, WTPdataR$Income_categorical_6cat, useNA = "ifany")

summary(WTPdataR)

library(psych)

describe(WTPdataR)

lapply(WTPdataR[sapply(WTPdataR, is.factor)], table)

prop.table(table(WTPdataR$Hadsmallermeals1)) * 100

table(WTPdataR$Hadsmallermeals1)

freq_table <- table(WTPdataR$Everbeenhungrybutnoteaten1)

percent_table <- prop.table(freq_table) * 100

data.frame(

Category = names(freq_table),

Count = as.vector(freq_table),

Percent = round(as.vector(percent_table), 1)

)

freq_table <- table(WTPdataR$Noteatenforawholeday1)

percent_table <- prop.table(freq_table) * 100

data.frame(

Category = names(freq_table),

Count = as.vector(freq_table),

Percent = round(as.vector(percent_table), 1)

)

freq_table <- table(WTPdataR$Noneofthese12)

percent_table <- prop.table(freq_table) * 100

data.frame(

Category = names(freq_table),

Count = as.vector(freq_table),

Percent = round(as.vector(percent_table), 1)

)

freq_table <- table(WTPdataR$Noneofthese1)

percent_table <- prop.table(freq_table) * 100

data.frame(

Category = names(freq_table),

Count = as.vector(freq_table),

Percent = round(as.vector(percent_table), 1)

)

freq_table <- table(WTPdataR$Hadsmallermeals12)

percent_table <- prop.table(freq_table) * 100

data.frame(

Category = names(freq_table),

Count = as.vector(freq_table),

Percent = round(as.vector(percent_table), 1)

)

freq_table <- table(WTPdataR$Everbeenhungrybutnoteaten12)

percent_table <- prop.table(freq_table) * 100

data.frame(

Category = names(freq_table),

Count = as.vector(freq_table),

Percent = round(as.vector(percent_table), 1)

)

freq_table <- table(WTPdataR$Noteatenforawholeday12)

percent_table <- prop.table(freq_table) * 100

data.frame(

Category = names(freq_table),

Count = as.vector(freq_table),

Percent = round(as.vector(percent_table), 1)

)

freq_table <- table(WTPdataR$AreyouWTP)

percent_table <- prop.table(freq_table) * 100

data.frame(

Category = names(freq_table),

Count = as.vector(freq_table),

Percent = round(as.vector(percent_table), 1)

)

freq_table <- table(WTPdataR$Gender)

percent_table <- prop.table(freq_table) * 100

data.frame(

Category = names(freq_table),

Count = as.vector(freq_table),

Percent = round(as.vector(percent_table), 1)

)

freq_table <- table(WTPdataR$Attendschbreakfastclub)

percent_table <- prop.table(freq_table) * 100

data.frame(

Category = names(freq_table),

Count = as.vector(freq_table),

Percent = round(as.vector(percent_table), 1)

)

freq_table <- table(WTPdataR$Payforbreakfastclub)

percent_table <- prop.table(freq_table) * 100

data.frame(

Category = names(freq_table),

Count = as.vector(freq_table),

Percent = round(as.vector(percent_table), 1)

)

freq_table <- table(WTPdataR$Freqofpayingbreakfast_4cat)

percent_table <- prop.table(freq_table) * 100

data.frame(

Category = names(freq_table),

Count = as.vector(freq_table),

Percent = round(as.vector(percent_table), 1)

)

freq_table <- table(WTPdataR$Typicallyeatlunch)

percent_table <- prop.table(freq_table) * 100

data.frame(

Category = names(freq_table),

Count = as.vector(freq_table),

Percent = round(as.vector(percent_table), 1)

)

freq_table <- table(WTPdataR$Familiaritywithlunchmenu)

percent_table <- prop.table(freq_table) * 100

data.frame(

Category = names(freq_table),

Count = as.vector(freq_table),

Percent = round(as.vector(percent_table), 1)

)

freq_table <- table(WTPdataR$Working)

percent_table <- prop.table(freq_table) * 100

data.frame(

Category = names(freq_table),

Count = as.vector(freq_table),

Percent = round(as.vector(percent_table), 1)

)

freq_table <- table(WTPdataR$Reception)

percent_table <- prop.table(freq_table) * 100

data.frame(

Category = names(freq_table),

Count = as.vector(freq_table),

Percent = round(as.vector(percent_table), 1)

)

freq_table <- table(WTPdataR$Year.1)

percent_table <- prop.table(freq_table) * 100

data.frame(

Category = names(freq_table),

Count = as.vector(freq_table),

Percent = round(as.vector(percent_table), 1)

)

freq_table <- table(WTPdataR$Year.2)

percent_table <- prop.table(freq_table) * 100

data.frame(

Category = names(freq_table),

Count = as.vector(freq_table),

Percent = round(as.vector(percent_table), 1)

)

freq_table <- table(WTPdataR$Year.3)

percent_table <- prop.table(freq_table) * 100

data.frame(

Category = names(freq_table),

Count = as.vector(freq_table),

Percent = round(as.vector(percent_table), 1)

)

freq_table <- table(WTPdataR$Year.4)

percent_table <- prop.table(freq_table) * 100

data.frame(

Category = names(freq_table),

Count = as.vector(freq_table),

Percent = round(as.vector(percent_table), 1)

)

freq_table <- table(WTPdataR$Year.5)

percent_table <- prop.table(freq_table) * 100

data.frame(

Category = names(freq_table),

Count = as.vector(freq_table),

Percent = round(as.vector(percent_table), 1)

)

freq_table <- table(WTPdataR$Year.6)

percent_table <- prop.table(freq_table) * 100

data.frame(

Category = names(freq_table),

Count = as.vector(freq_table),

Percent = round(as.vector(percent_table), 1)

)

freq_table <- table(WTPdataR$Ethnicity)

percent_table <- prop.table(freq_table) * 100

data.frame(

Category = names(freq_table),

Count = as.vector(freq_table),

Percent = round(as.vector(percent_table), 1)

)

freq_table <- table(WTPdataR$UK.regions)

percent_table <- prop.table(freq_table) * 100

data.frame(

Category = names(freq_table),

Count = as.vector(freq_table),

Percent = round(as.vector(percent_table), 1)

)

WTPdataR$Noneofthese1_rev <- ifelse(WTPdataR$Noneofthese1 == 1, 0, 1)

WTPdataR$FoodInsecurityIndex <- rowSums(

WTPdataR[, c("Hadsmallermeals1",

"Everbeenhungrybutnoteaten1",

"Noteatenforawholeday1",

"Noneofthese1_rev")],

na.rm = TRUE

)

WTPdataR$Noneofthese12_rev <- ifelse(WTPdataR$Noneofthese12 == 1, 0, 1)

WTPdataR$FoodInsecurityIndex12 <- rowSums(

WTPdataR[, c("Hadsmallermeals12",

"Everbeenhungrybutnoteaten12",

"Noteatenforawholeday12",

"Noneofthese12_rev")],

na.rm = TRUE

)

"Hadsmallermeals12" %in% names(WTPdataR)

"Everbeenhungrybutnoteaten12" %in% names(WTPdataR)

"Noteatenforawholeday12" %in% names(WTPdataR)

"Noneofthese12_rev" %in% names(WTPdataR)

# Example if names are correct

WTPdataR$Noneofthese12_rev <- ifelse(WTPdataR$Noneofthese12 == 1, 0, 1)

WTPdataR$FoodInsecurityIndex12 <- rowSums(

WTPdataR[, c("Hadsmallermeals12",

"Everbeenhungrybutnoteaten12",

"Noteatenforawholeday12",

"Noneofthese12_rev")],

na.rm = TRUE

)

freq_table <- table(WTPdataR$FoodInsecurityIndex)

percent_table <- prop.table(freq_table) * 100

data.frame(

Category = names(freq_table),

Count = as.vector(freq_table),

Percent = round(as.vector(percent_table), 1)

)

freq_table <- table(WTPdataR$FoodInsecurityIndex12)

percent_table <- prop.table(freq_table) * 100

data.frame(

Category = names(freq_table),

Count = as.vector(freq_table),

Percent = round(as.vector(percent_table), 1)

)

hist(WTPdataR$WTP.1,

breaks = 30,

main = "Willingness to Pay for a Standard School Lunch",

xlab = "Willingness to Pay (£)",

ylab = "Frequency",

col = "skyblue",

border = NA)

abline(v = mean(WTPdataR$WTP.1, na.rm = TRUE), col = "red", lwd = 2)

hist(WTPdataR$WTP.2,

breaks = 30,

main = "Willingness to Pay for a Nutritious School Lunch",

xlab = "Willingness to Pay (£)",

ylab = "Frequency",

col = "lightgreen",

border = NA)

abline(v = mean(WTPdataR$WTP.2, na.rm = TRUE), col = "red", lwd = 2)

# Overlayed comparison plot

# Compute density estimates

# Compute density for WTP.1

d1 <- density(WTPdataR$WTP.1, na.rm = TRUE)

# Plot WTP.1 (Standard School Lunch)

plot(d1,

main = "Willingness to Pay for a Standard School Lunch",

xlab = "Willingness to Pay (£)",

ylab = "Density",

col = NA,

lwd = 2,

cex.main = 1.2,

cex.lab = 1,

ylim = c(0, max(d1$y) * 1.1)) # add small margin for aesthetics

# Add shaded area and density line

polygon(d1, col = rgb(0.2, 0.6, 0.9, 0.4), border = NA)

lines(d1, col = "skyblue4", lwd = 2)

# Add mean line

abline(v = mean(WTPdataR$WTP.1, na.rm = TRUE), col = "red", lwd = 2, lty = 2)

# Optional legend

legend("topright",

legend = c("Density", "Mean WTP"),

col = c("skyblue4", "red"),

lwd = c(2, 2),

lty = c(1, 2),

bty = "n")

# Compute density for WTP.2

d2 <- density(WTPdataR$WTP.2, na.rm = TRUE)

# Plot WTP.2 (Nutritious School Lunch)

plot(d2,

main = "Willingness to Pay for a Nutritious School Lunch",

xlab = "Willingness to Pay (£)",

ylab = "Density",

col = NA,

lwd = 2,

cex.main = 1.2,

cex.lab = 1,

ylim = c(0, max(d2$y) * 1.1))

# Add shaded area and density line

polygon(d2, col = rgb(0.1, 0.9, 0.1, 0.4), border = NA)

lines(d2, col = "forestgreen", lwd = 2)

# Add mean line

abline(v = mean(WTPdataR$WTP.2, na.rm = TRUE), col = "red", lwd = 2, lty = 2)

# Optional legend

legend("topright",

legend = c("Density", "Mean WTP"),

col = c("forestgreen", "red"),

lwd = c(2, 2),

lty = c(1, 2),

bty = "n")

describe(WTPdataR$WTP.1)

describe(WTPdataR$WTP.2)

# Model 1 – Only Gender

WTPdataR$Gender <- factor(WTPdataR$Gender,

levels = c(1, 2),

labels = c("1", "2"))

WTPdataR$Gender <- relevel(WTPdataR$Gender, ref = "1")

mod1 <- lm(WTP.1 ~ Gender, data = WTPdataR)

summary(mod1)

mod2 <- lm(WTP.1 ~ Gender + Age_continuous_num, data = WTPdataR)

summary(mod2)

mod3 <- lm(WTP.1 ~ Gender + Age_continuous_num + Income_continuous_num, data = WTPdataR)

summary(mod3)

mod4 <- lm(WTP.1 ~ Gender + Age_continuous_num + Income_continuous_num + AdultHHsize_cont, data = WTPdataR)

summary(mod4)

WTPdataR$FoodInsecurityIndex <- factor(WTPdataR$FoodInsecurityIndex,

levels = c(0, 1, 2, 3, 4),

labels = c("0", "1", "2", "3", "4"))

WTPdataR$FoodInsecurityIndex <- relevel(WTPdataR$FoodInsecurityIndex, ref = "0")

mod5 <- lm(WTP.1 ~ Gender + Age_continuous_num + Income_continuous_num + AdultHHsize_cont + FoodInsecurityIndex, data = WTPdataR)

summary(mod5)

library(stringr)

WTPdataR$Income_continuous_num <- sapply(WTPdataR$Income_continuous, function(x) {

x <- trimws(x) # remove spaces

if (grepl("to", x, ignore.case = TRUE)) {

# Extract all numeric parts, remove commas, and take their mean

nums <- as.numeric(gsub(",", "", unlist(str_extract_all(x, "[0-9]+"))))

mean(nums) / 10000

} else if (grepl("Under", x, ignore.case = TRUE)) {

1 # represent "Under ..." as roughly £10,000 → 1 unit of £10,000

} else if (grepl("Don't know|Prefer not", x, ignore.case = TRUE)) {

NA

} else if (grepl("[0-9]", x)) {

# For single number cases like "150,000 and over"

as.numeric(gsub(",", "", str_extract(x, "[0-9]+"))) / 10000

} else {

NA

}

})

WTPdataR$Income_continuous[1:10]

x <- "70,000 to 99,999 per year"

as.numeric(gsub(",", "", str_extract(x, "[0-9]+")))

library(stringr)

WTPdataR$Income_continuous_num <- sapply(WTPdataR$Income_continuous, function(x) {

x <- trimws(x)

if (grepl("to", x, ignore.case = TRUE)) {

# Extract both numbers properly (with commas)

nums <- as.numeric(gsub(",", "", unlist(str_extract_all(x, "\\d{1,3}(?:,\\d{3})*"))))

mean(nums) / 10000

} else if (grepl("Under", x, ignore.case = TRUE)) {

1

} else if (grepl("Don't know|Prefer not", x, ignore.case = TRUE)) {

NA

} else if (grepl("[0-9]", x)) {

# For single number cases like "150,000 and over"

as.numeric(gsub(",", "", str_extract(x, "\\d{1,3}(?:,\\d{3})*"))) / 10000

} else {

NA

}

})

WTPdataR$FoodInsecurityIndex12 <- factor(WTPdataR$FoodInsecurityIndex12,

levels = c(0, 1, 2, 3, 4),

labels = c("0", "1", "2", "3", "4"))

WTPdataR$FoodInsecurityIndex12 <- relevel(WTPdataR$FoodInsecurityIndex12, ref = "0")

mod6 <- lm(WTP.1 ~ Gender + Age_continuous_num + Income_continuous_num + AdultHHsize_cont + FoodInsecurityIndex + FoodInsecurityIndex12, data = WTPdataR)

summary(mod6)

WTPdataR$Attendschbreakfastclub <- factor(WTPdataR$Attendschbreakfastclub,

levels = c(0, 1),

labels = c("0", "1"))

WTPdataR$Attendschbreakfastclub <- relevel(WTPdataR$Attendschbreakfastclub, ref = "0")

mod7 <- lm(WTP.1 ~ Gender + Age_continuous_num + Income_continuous_num + AdultHHsize_cont + FoodInsecurityIndex + FoodInsecurityIndex12 + Attendschbreakfastclub, data = WTPdataR)

summary(mod7)

WTPdataR$Payforbreakfastclub <- factor(WTPdataR$Payforbreakfastclub,

levels = c(0, 1),

labels = c("0", "1"))

WTPdataR$Payforbreakfastclub <- relevel(WTPdataR$Payforbreakfastclub, ref = "0")

mod8 <- lm(WTP.1 ~ Gender + Age_continuous_num + Income_continuous_num + AdultHHsize_cont + FoodInsecurityIndex + FoodInsecurityIndex12 + Attendschbreakfastclub + Payforbreakfastclub, data = WTPdataR)

summary(mod8)

sapply(WTPdataR[, c("Gender", "Age_continuous_num", "Income_continuous_num",

"AdultHHsize_cont", "FoodInsecurityIndex", "FoodInsecurityIndex12",

"Attendschbreakfastclub", "Payforbreakfastclub")],

function(x) length(unique(na.omit(x))))

complete_data <- na.omit(WTPdataR[, c("WTP.1", "Gender", "Age_continuous_num",

"Income_continuous_num", "AdultHHsize_cont",

"FoodInsecurityIndex", "FoodInsecurityIndex12",

"Attendschbreakfastclub", "Payforbreakfastclub")])

sapply(complete_data, function(x) length(unique(x)))

table(complete_data$Attendschbreakfastclub, useNA = "ifany")

table(WTPdataR$Payforbreakfastclub, useNA = "ifany")

table(WTPdataR$Freqofpayingbreakfast_4cat, useNA = "ifany")

WTPdataR$Typicallyeatlunch <- factor(WTPdataR$Typicallyeatlunch,

levels = c(1, 2, 3),

labels = c("1", "2", "3"))

WTPdataR$Typicallyeatlunch <- relevel(WTPdataR$Typicallyeatlunch, ref = "1")

mod9 <- lm(WTP.1 ~ Gender + Age_continuous_num + Income_continuous_num + AdultHHsize_cont + FoodInsecurityIndex + FoodInsecurityIndex12 + Attendschbreakfastclub + Typicallyeatlunch, data = WTPdataR)

summary(mod9)

table(WTPdataR$Familiaritywithlunchmenu, useNA = "ifany")

WTPdataR$Familiaritywithlunchmenu <- factor(WTPdataR$Familiaritywithlunchmenu,

levels = c(0, 1),

labels = c("0", "1"))

WTPdataR$Familiaritywithlunchmenu <- relevel(WTPdataR$Familiaritywithlunchmenu, ref = "0")

mod10 <- lm(WTP.1 ~ Gender + Age_continuous_num + Income_continuous_num + AdultHHsize_cont + FoodInsecurityIndex + FoodInsecurityIndex12 + Attendschbreakfastclub + Typicallyeatlunch + Familiaritywithlunchmenu, data = WTPdataR)

summary(mod10)

WTPdataR$Perceptionofschlunch_3cat <- factor(WTPdataR$Perceptionofschlunch_3cat,

levels = c(1, 2, 3),

labels = c("1", "2", "3"))

WTPdataR$Perceptionofschlunch_3cat <- relevel(WTPdataR$Perceptionofschlunch_3cat, ref = "1")

mod11 <- lm(WTP.1 ~ Gender + Age_continuous_num + Income_continuous_num + AdultHHsize_cont + FoodInsecurityIndex + FoodInsecurityIndex12 + Attendschbreakfastclub + Typicallyeatlunch + Perceptionofschlunch_3cat, data = WTPdataR)

summary(mod11)

WTPdataR$Working <- factor(WTPdataR$Working,

levels = c(0, 1),

labels = c("0", "1"))

WTPdataR$Working <- relevel(WTPdataR$Working, ref = "0")

mod12 <- lm(WTP.1 ~ Gender + Age_continuous_num + Income_continuous_num + AdultHHsize_cont + FoodInsecurityIndex + FoodInsecurityIndex12 + Attendschbreakfastclub + Typicallyeatlunch + Perceptionofschlunch_3cat + Working, data = WTPdataR)

summary(mod12)

WTPdataR$Ethnicity <- factor(WTPdataR$Ethnicity,

levels = c(1, 2),

labels = c("1", "2"))

WTPdataR$Ethnicity <- relevel(WTPdataR$Ethnicity, ref = "1")

mod13 <- lm(WTP.1 ~ Gender + Age_continuous_num + Income_continuous_num + AdultHHsize_cont + FoodInsecurityIndex + FoodInsecurityIndex12 + Attendschbreakfastclub + Typicallyeatlunch + Perceptionofschlunch_3cat + Working + Ethnicity, data = WTPdataR)

summary(mod13)

WTPdataR$UK.regions<- factor(WTPdataR$UK.regions,

levels = c(1, 2, 3, 4, 5, 6, 7, 8, 9),

labels = c("1", "2", "3", "4", "5", "6", "7", "8", "9"))

WTPdataR$UK.regions <- relevel(WTPdataR$UK.regions, ref = "7")

mod14 <- lm(WTP.1 ~ Gender + Age_continuous_num + Income_continuous_num + AdultHHsize_cont + FoodInsecurityIndex + FoodInsecurityIndex12 + Attendschbreakfastclub + Typicallyeatlunch + Perceptionofschlunch_3cat + Working + Ethnicity + UK.regions, data = WTPdataR)

summary(mod14)

WTPdataR$Reception <- factor(WTPdataR$Reception,

levels = c(0, 1),

labels = c("0", "1"))

WTPdataR$Reception <- relevel(WTPdataR$Reception, ref = "0")

mod15 <- lm(WTP.1 ~ Gender + Age_continuous_num + Income_continuous_num + AdultHHsize_cont + FoodInsecurityIndex + FoodInsecurityIndex12 + Attendschbreakfastclub + Typicallyeatlunch + Perceptionofschlunch_3cat + Working + Ethnicity + UK.regions + Reception, data = WTPdataR)

summary(mod15)

WTPdataR$Year.1<- factor(WTPdataR$Year.1,

levels = c(0, 1),

labels = c("0", "1"))

WTPdataR$Year.1 <- relevel(WTPdataR$Year.1, ref = "0")

mod16 <- lm(WTP.1 ~ Gender + Age_continuous_num + Income_continuous_num + AdultHHsize_cont + FoodInsecurityIndex + FoodInsecurityIndex12 + Attendschbreakfastclub + Typicallyeatlunch + Perceptionofschlunch_3cat + Working + Ethnicity + UK.regions + Reception +Year.1, data = WTPdataR)

summary(mod16)

WTPdataR$Year.2<- factor(WTPdataR$Year.2,

levels = c(0, 1),

labels = c("0", "1"))

WTPdataR$Year.2 <- relevel(WTPdataR$Year.2, ref = "0")

mod17 <- lm(WTP.1 ~ Gender + Age_continuous_num + Income_continuous_num + AdultHHsize_cont + FoodInsecurityIndex + FoodInsecurityIndex12 + Attendschbreakfastclub + Typicallyeatlunch + Perceptionofschlunch_3cat + Working + Ethnicity + UK.regions + Reception +Year.1 + Year.2, data = WTPdataR)

summary(mod17)

WTPdataR$Year.3<- factor(WTPdataR$Year.3,

levels = c(0, 1),

labels = c("0", "1"))

WTPdataR$Year.3 <- relevel(WTPdataR$Year.3, ref = "0")

mod18 <- lm(WTP.1 ~ Gender + Age_continuous_num + Income_continuous_num + AdultHHsize_cont + FoodInsecurityIndex + FoodInsecurityIndex12 + Attendschbreakfastclub + Typicallyeatlunch + Perceptionofschlunch_3cat + Working + Ethnicity + UK.regions + Reception +Year.1 + Year.2 + Year.3, data = WTPdataR)

summary(mod18)

WTPdataR$Year.4<- factor(WTPdataR$Year.4,

levels = c(0, 1),

labels = c("0", "1"))

WTPdataR$Year.4 <- relevel(WTPdataR$Year.4, ref = "0")

mod19 <- lm(WTP.1 ~ Gender + Age_continuous_num + Income_continuous_num + AdultHHsize_cont + FoodInsecurityIndex + FoodInsecurityIndex12 + Attendschbreakfastclub + Typicallyeatlunch + Perceptionofschlunch_3cat + Working + Ethnicity + UK.regions + Reception +Year.1 + Year.2 + Year.3 + Year.4, data = WTPdataR)

summary(mod19)

WTPdataR$Year.5<- factor(WTPdataR$Year.5,

levels = c(0, 1),

labels = c("0", "1"))

WTPdataR$Year.5 <- relevel(WTPdataR$Year.5, ref = "0")

mod20 <- lm(WTP.1 ~ Gender + Age_continuous_num + Income_continuous_num + AdultHHsize_cont + FoodInsecurityIndex + FoodInsecurityIndex12 + Attendschbreakfastclub + Typicallyeatlunch + Perceptionofschlunch_3cat + Working + Ethnicity + UK.regions + Reception +Year.1 + Year.2 + Year.3 + Year.4 + Year.5, data = WTPdataR)

summary(mod20)

WTPdataR$Year.6<- factor(WTPdataR$Year.6,

levels = c(0, 1),

labels = c("0", "1"))

WTPdataR$Year.6 <- relevel(WTPdataR$Year.6, ref = "0")

mod21 <- lm(WTP.1 ~ Gender + Age_continuous_num + Income_continuous_num + AdultHHsize_cont + FoodInsecurityIndex + FoodInsecurityIndex12 + Attendschbreakfastclub + Typicallyeatlunch + Perceptionofschlunch_3cat + Working + Ethnicity + UK.regions + Reception +Year.1 + Year.2 + Year.3 + Year.4 + Year.5 + Year.6, data = WTPdataR)

summary(mod21)

mod16 <- lm(WTP.1 ~ Gender + Age_continuous_num + Income_continuous_num + AdultHHsize_cont + FoodInsecurityIndex + FoodInsecurityIndex12 + Attendschbreakfastclub + Typicallyeatlunch + Perceptionofschlunch_3cat + Working + Ethnicity + UK.regions + Reception +Year.1, data = WTPdataR)

summary(mod16)

plot(mod16, which = 1) # Residuals vs Fitted

library(lmtest)

bptest(mod16) # Breusch-Pagan test

plot(mod16, which = 2) # Q-Q plot

shapiro.test(residuals(mod16))

library(car)

vif(mod16)

AIC(mod5, mod6, mod11, mod14, mod13, mod24)

#Fit a simple regression for age only

mod_age <- lm(WTP.1 ~ Age_continuous_num, data = WTPdataR)

#Component + residual plot (visual linearity check)

library(car)

crPlots(mod_age)

#Box–Tidwell test (statistical linearity check)

boxTidwell(WTP.1 ~ Age_continuous_num, data = WTPdataR)

mod_income <- lm(WTP.1 ~ Income_continuous_num, data = WTPdataR)

crPlots(mod_income)

boxTidwell(WTP.1 ~ Income_continuous_num, data = WTPdataR)

mod_house <- lm(WTP.1 ~ AdultHHsize_cont, data = WTPdataR)

crPlots(mod_house)

boxTidwell(WTP.1 ~ AdultHHsize_cont, data = WTPdataR)

library(sandwich)

library(lmtest)

# Robust (HC1) standard errors and adjusted t-tests

coeftest(mod16, vcov = vcovHC(mod16, type = "HC1"))

AIC(mod21)

install.packages("stargazer") #Must be connected to the internet

library(stargazer)

stargazer(mod5, mod6, mod14, mod16) #For use in Latex

library(stargazer)

library(lmtest)

library(sandwich)

# Compute robust SE for mod16

robust_se_mod16 <- sqrt(diag(vcovHC(mod16, type = "HC1")))

# Generate the table

stargazer(mod5, mod6, mod11, mod16,

se = list(NULL, NULL, NULL, robust_se_mod16),

type = "latex",

title = "Willingness to Pay for the cost of a standard school lunch",

dep.var.labels = c("Willingness to pay for the cost of a standard school lunch"),

column.labels = c("(model 1)", "(model 2)", "(model 3)", "(Final model 4)"),

align = TRUE,

digits = 3,

omit.stat = c("f", "ser"),

star.cutoffs = c(0.1, 0.05, 0.01),

notes = "Robust (HC1) standard errors in parentheses for Model 4 only.",

notes.align = "l")

modint1 <- lm(WTP.1 ~ Income_continuous_num * FoodInsecurityIndex, data = WTPdataR)

summary(modint1)

modint2 <- lm(WTP.1 ~ Income_continuous_num * FoodInsecurityIndex, data = WTPdataR)

summary(modint2)

modint3 <- lm(WTP.1 ~ Income_continuous_num * FoodInsecurityIndex12, data = WTPdataR)

summary(modint3)

modint4 <- lm(WTP.1 ~ AdultHHsize_cont * Income_continuous_num, data = WTPdataR)

summary(modint4)

modint5 <- lm(WTP.1 ~ FoodInsecurityIndex * Attendschbreakfastclub, data = WTPdataR)

summary(modint5)

modint6 <- lm(WTP.1 ~ FoodInsecurityIndex12 * Attendschbreakfastclub, data = WTPdataR)

summary(modint6)

modint7 <- lm(WTP.1 ~ Perceptionofschlunch_3cat * Typicallyeatlunch, data = WTPdataR)

summary(modint7)

modint8 <- lm(WTP.1 ~ Gender * FoodInsecurityIndex, data = WTPdataR)

summary(modint8)

modint9 <- lm(WTP.1 ~ Gender * FoodInsecurityIndex12, data = WTPdataR)

summary(modint9)

mod22 <- lm(WTP.1 ~ Gender + Age_continuous_num + Income_continuous_num * AdultHHsize_cont +

FoodInsecurityIndex + FoodInsecurityIndex12 +

Gender:FoodInsecurityIndex +

Perceptionofschlunch_3cat * Typicallyeatlunch +

Attendschbreakfastclub + Working + Ethnicity + UK.regions +

Reception + Year.1,

data = WTPdataR)

summary(mod22)

AIC(mod22)

mod23 <- lm(WTP.1 ~ Gender + Age_continuous_num + Income_continuous_num * AdultHHsize_cont +

FoodInsecurityIndex + FoodInsecurityIndex12 +

Perceptionofschlunch_3cat * Typicallyeatlunch +

Attendschbreakfastclub + Working + Ethnicity + UK.regions +

Reception + Year.1,

data = WTPdataR)

summary(mod23)

AIC(mod23)

# Compute robust SE for mod16

robust_se_mod23 <- sqrt(diag(vcovHC(mod23, type = "HC1")))

# Generate the table

stargazer(mod6, mod11, mod16, mod23)

se = list(NULL, NULL, NULL, robust_se_mod23)

type = "latex"

title = "Willingness to Pay for the cost of a standard school lunch"

dep.var.labels = c("Willingness to pay for the cost of a standard school lunch")

column.labels = c("(model 1)", "(model 2)", "(model 3)", "(Final model 4)")

align = TRUE

digits = 3

omit.stat = c("f", "ser")

star.cutoffs = c(0.1, 0.05, 0.01)

notes = "Robust (HC1) standard errors in parentheses for Model 4 only."

notes.align = "l"

mod_4 <- lm(WTP.2 ~ Gender + Age_continuous_num + Income_continuous_num + AdultHHsize_cont, data = WTPdataR)

summary(mod_4)

mod_5 <- lm(WTP.2 ~ Gender + Age_continuous_num + Income_continuous_num + AdultHHsize_cont + FoodInsecurityIndex, data = WTPdataR)

summary(mod_5)

mod_6 <- lm(WTP.2 ~ Gender + Age_continuous_num + Income_continuous_num + AdultHHsize_cont + FoodInsecurityIndex + FoodInsecurityIndex12, data = WTPdataR)

summary(mod_6)

mod_7 <- lm(WTP.2 ~ Gender + Age_continuous_num + Income_continuous_num + AdultHHsize_cont + FoodInsecurityIndex + FoodInsecurityIndex12 + Attendschbreakfastclub, data = WTPdataR)

summary(mod_7)

mod_9 <- lm(WTP.2 ~ Gender + Age_continuous_num + Income_continuous_num + AdultHHsize_cont + FoodInsecurityIndex + FoodInsecurityIndex12 + Attendschbreakfastclub + Typicallyeatlunch, data = WTPdataR)

summary(mod_9)

mod_11 <- lm(WTP.2 ~ Gender + Age_continuous_num + Income_continuous_num + AdultHHsize_cont + FoodInsecurityIndex + FoodInsecurityIndex12 + Attendschbreakfastclub + Typicallyeatlunch + Perceptionofschlunch_3cat, data = WTPdataR)

summary(mod_11)

mod_12 <- lm(WTP.2 ~ Gender + Age_continuous_num + Income_continuous_num + AdultHHsize_cont + FoodInsecurityIndex + FoodInsecurityIndex12 + Attendschbreakfastclub + Typicallyeatlunch + Perceptionofschlunch_3cat + Working, data = WTPdataR)

summary(mod_12)

mod_13 <- lm(WTP.2 ~ Gender + Age_continuous_num + Income_continuous_num + AdultHHsize_cont + FoodInsecurityIndex + FoodInsecurityIndex12 + Attendschbreakfastclub + Typicallyeatlunch + Perceptionofschlunch_3cat + Working + Ethnicity, data = WTPdataR)

summary(mod_13)

mod_14 <- lm(WTP.2 ~ Gender + Age_continuous_num + Income_continuous_num + AdultHHsize_cont + FoodInsecurityIndex + FoodInsecurityIndex12 + Attendschbreakfastclub + Typicallyeatlunch + Perceptionofschlunch_3cat + Working + Ethnicity + UK.regions, data = WTPdataR)

summary(mod_14)

mod_15 <- lm(WTP.2 ~ Gender + Age_continuous_num + Income_continuous_num + AdultHHsize_cont + FoodInsecurityIndex + FoodInsecurityIndex12 + Attendschbreakfastclub + Typicallyeatlunch + Perceptionofschlunch_3cat + Working + Ethnicity + UK.regions + Reception, data = WTPdataR)

summary(mod_15)

mod_16 <- lm(WTP.2 ~ Gender + Age_continuous_num + Income_continuous_num + AdultHHsize_cont + FoodInsecurityIndex + FoodInsecurityIndex12 + Attendschbreakfastclub + Typicallyeatlunch + Perceptionofschlunch_3cat + Working + Ethnicity + UK.regions + Reception +Year.1, data = WTPdataR)

summary(mod_16)

mod_17 <- lm(WTP.2 ~ Gender + Age_continuous_num + Income_continuous_num + AdultHHsize_cont + FoodInsecurityIndex + FoodInsecurityIndex12 + Attendschbreakfastclub + Typicallyeatlunch + Perceptionofschlunch_3cat + Working + Ethnicity + UK.regions + Reception +Year.1 + Year.2, data = WTPdataR)

summary(mod_17)

mod_18 <- lm(WTP.2 ~ Gender + Age_continuous_num + Income_continuous_num + AdultHHsize_cont + FoodInsecurityIndex + FoodInsecurityIndex12 + Attendschbreakfastclub + Typicallyeatlunch + Perceptionofschlunch_3cat + Working + Ethnicity + UK.regions + Reception +Year.1 + Year.2 + Year.3, data = WTPdataR)

summary(mod_18)

mod_19 <- lm(WTP.2 ~ Gender + Age_continuous_num + Income_continuous_num + AdultHHsize_cont + FoodInsecurityIndex + FoodInsecurityIndex12 + Attendschbreakfastclub + Typicallyeatlunch + Perceptionofschlunch_3cat + Working + Ethnicity + UK.regions + Reception +Year.1 + Year.2 + Year.3 + Year.4, data = WTPdataR)

summary(mod_19)

mod_20 <- lm(WTP.2 ~ Gender + Age_continuous_num + Income_continuous_num + AdultHHsize_cont + FoodInsecurityIndex + FoodInsecurityIndex12 + Attendschbreakfastclub + Typicallyeatlunch + Perceptionofschlunch_3cat + Working + Ethnicity + UK.regions + Reception +Year.1 + Year.2 + Year.3 + Year.4 + Year.5, data = WTPdataR)

summary(mod_20)

mod_21 <- lm(WTP.2 ~ Gender + Age_continuous_num + Income_continuous_num + AdultHHsize_cont + FoodInsecurityIndex + FoodInsecurityIndex12 + Attendschbreakfastclub + Typicallyeatlunch + Perceptionofschlunch_3cat + Working + Ethnicity + UK.regions + Reception +Year.1 + Year.2 + Year.3 + Year.4 + Year.5 + Year.6, data = WTPdataR)

summary(mod_21)

mod_int1 <- lm(WTP.2 ~ Income_continuous_num * FoodInsecurityIndex, data = WTPdataR)

summary(mod_int1)

mod_int2 <- lm(WTP.2 ~ Income_continuous_num * FoodInsecurityIndex, data = WTPdataR)

summary(mod_int2)

mod_int3 <- lm(WTP.2 ~ Income_continuous_num * FoodInsecurityIndex12, data = WTPdataR)

summary(mod_int3)

mod_int4 <- lm(WTP.2 ~ AdultHHsize_cont * Income_continuous_num, data = WTPdataR)

summary(mod_int4)

mod_int5 <- lm(WTP.2 ~ FoodInsecurityIndex * Attendschbreakfastclub, data = WTPdataR)

summary(mod_int5)

mod_int6 <- lm(WTP.2 ~ FoodInsecurityIndex12 * Attendschbreakfastclub, data = WTPdataR)

summary(mod_int6)

mod_int7 <- lm(WTP.2 ~ Perceptionofschlunch_3cat * Typicallyeatlunch, data = WTPdataR)

summary(mod_int7)

mod_int8 <- lm(WTP.2 ~ Gender * FoodInsecurityIndex, data = WTPdataR)

summary(mod_int8)

mod_int9 <- lm(WTP.2 ~ Gender * FoodInsecurityIndex12, data = WTPdataR)

summary(mod_int9)

AIC(mod_5, mod_6, mod_9, mod_11, mod_13)

mod24 <- lm(WTP.1 ~ Gender + Age_continuous_num + Income_continuous_num * AdultHHsize_cont +

FoodInsecurityIndex + FoodInsecurityIndex12 +

Perceptionofschlunch_3cat * Typicallyeatlunch +

Attendschbreakfastclub + Working + Ethnicity,

data = WTPdataR)

summary(mod24)

AIC(mod24)

WTPdataR$Predicted_WTP1 <- predict(mod24, newdata = WTPdataR)

summary(WTPdataR$Predicted_WTP1)

# For WTP.2 model (replace 'mod_nutrious' with the name of your nutritious-lunch model)

WTPdataR$Predicted_WTP2 <- predict(mod_13, newdata = WTPdataR)

summary(WTPdataR$Predicted_WTP2)

stargazer(mod24, mod_13)

coeftest(mod24, vcov = vcovHC(mod16, type = "HC1"))

# Compute robust (HC1) variance–covariance matrix

robust_se_mod24 <- vcovHC(mod24, type = "HC1")

# Generate coefficient table with robust SEs

robust_test_mod24 <- coeftest(mod24, vcov = robust_se_mod24)

# View results in R console

robust_test_mod24

# Compute robust (HC1) variance–covariance matrix

robust_se_mod_13 <- vcovHC(mod_13, type = "HC1")

# Generate coefficient table with robust SEs

robust_test_mod_13 <- coeftest(mod_13, vcov = robust_se_mod_13)

# View results in R console

robust_test_mod_13

# Create Stargazer table with robust SEs

stargazer(mod24, mod_13,

se = list(sqrt(diag(robust_se_mod24)),

sqrt(diag(robust_se_mod_13))),

title = "Comparison of Willingness to Pay Models",

align = TRUE,

header = FALSE,

dep.var.labels = c("Willingness to Pay (£)", "WTP.2"),

column.labels = c("Model 24 (Final, Robust)", "Model 13"),

covariate.labels = c(

# --- Demographics ---

"Female",

"Age (years)",

"Income (× £10,000)",

"Adult household size",

# --- Food Insecurity (current) ---

"Moderate Food Insecurity",

"Severe Food Insecurity",

"Very Severe Food Insecurity",

# --- Food Insecurity (12 months) ---

"Mild Food Insecurity (12 months)",

"Moderate Food Insecurity (12 months)",

"Severe Food Insecurity (12 months)",

"Very Severe Food Insecurity (12 months)",

# --- Perception and lunch pattern ---

"Perception of school lunch (Medium)",

"Perception of school lunch (Low/Not sure)",

"Typically eats school lunch (Sometimes)",

"Typically eats school lunch (Mostly)",

# --- Other predictors ---

"Attends school breakfast club",

"Working",

"Ethnicity (non-White)",

# --- Interactions (for Model 24 only) ---

"Income × Household size",

"Perception × Typical lunch"

),

omit.stat = c("f", "ser"),

notes = c("Robust (HC1) standard errors in parentheses for both models.",

"* p < 0.1; ** p < 0.05; *** p < 0.01"),

notes.align = "l",

type = "latex",

single.row = FALSE, # SEs on separate line

no.space = FALSE, # Adds space between rows

font.size = "small") # Keeps everything compact but readable

library(ggplot2)

# Calculate means and 95% CI

means_df <- data.frame(

LunchType = c("Standard Lunch", "Nutritious Lunch"),

Mean = c(mean(WTPdataR$Predicted_WTP1, na.rm = TRUE),

mean(WTPdataR$Predicted_WTP2, na.rm = TRUE)),

SD = c(sd(WTPdataR$Predicted_WTP1, na.rm = TRUE),

sd(WTPdataR$Predicted_WTP2, na.rm = TRUE)),

N = c(sum(!is.na(WTPdataR$Predicted_WTP1)),

sum(!is.na(WTPdataR$Predicted_WTP2)))

)

means_df$SE <- means_df$SD / sqrt(means_df$N)

means_df$CI_low <- means_df$Mean - 1.96 * means_df$SE

means_df$CI_high <- means_df$Mean + 1.96 * means_df$SE

ggplot(means_df, aes(x = LunchType, y = Mean, fill = LunchType)) +

geom_bar(stat = "identity", width = 0.6, colour = "black") +

geom_errorbar(aes(ymin = CI_low, ymax = CI_high), width = 0.15) +

labs(title = "Mean Predicted Willingness to Pay",

x = NULL, y = "Predicted WTP (£)") +

scale_fill_manual(values = c("#5DADE2", "#58D68D")) +

theme_minimal(base_size = 14) +

theme(legend.position = "none")

library(dplyr)

library(ggplot2)

library(tidyr)

# Combine both predicted values

WTP_long <- WTPdataR %>%

select(Predicted_WTP1, Predicted_WTP2) %>%

pivot_longer(cols = everything(),

names_to = "LunchType",

values_to = "Predicted_WTP")

# Clean labels

WTP_long$LunchType <- factor(WTP_long$LunchType,

labels = c("Standard Lunch", "Nutritious Lunch"))

# Plot

ggplot(WTP_long, aes(x = Predicted_WTP, fill = LunchType)) +

geom_density(alpha = 0.4) +

labs(title = "Distribution of Predicted Willingness to Pay",

x = "Predicted WTP (£)",

y = "Density",

fill = "Lunch Type") +

scale_fill_manual(values = c("#3498DB", "#2ECC71")) +

theme_minimal(base_size = 14)

install.packages("psych") # Run once if not installed

library(psych)

describe(WTPdataR$Age_continuous_num)

stargazer(mod5, mod6, mod11, mod13)

stargazer(mod_5, mod_6, mod_9, mod_11)

table(WTPdataR$AreyouWTP)

WTPdataR$NotWTP <- ifelse(WTPdataR$AreyouWTP == 1, 0, 1)

table(WTPdataR$NotWTP)

logit_modd1 <- glm(NotWTP ~ Gender, data = WTPdataR, family = binomial)

# Extract coefficients and standard errors

coefs <- summary(logit_modd1)$coefficients

# Combine with odds ratios and confidence intervals

results <- cbind(

Odds_Ratio = exp(coef(logit_modd1)),

SE = coefs[, "Std. Error"],

z_value = coefs[, "z value"],

p_value = coefs[, "Pr(>|z|)"],

Lower_95CI = exp(confint(logit_modd1))[, 1],

Upper_95CI = exp(confint(logit_modd1))[, 2]

)

# View tidy table

round(results, 3)

logit_modd2 <- glm(NotWTP ~ Gender + Age_continuous_num, data = WTPdataR, family = binomial)

summary(logit_modd2)

coefs <- summary(logit_modd2)$coefficients

# Combine with odds ratios and confidence intervals

results <- cbind(

Odds_Ratio = exp(coef(logit_modd2)),

SE = coefs[, "Std. Error"],

z_value = coefs[, "z value"],

p_value = coefs[, "Pr(>|z|)"],

Lower_95CI = exp(confint(logit_modd2))[, 1],

Upper_95CI = exp(confint(logit_modd2))[, 2]

)

# View tidy table

round(results, 3)

logit_modd3 <- glm(NotWTP ~ Gender + Age_continuous_num + Income_continuous_num + AdultHHsize_cont,

data = WTPdataR, family = binomial)

summary(logit_modd3)

coefs <- summary(logit_modd3)$coefficients

# Combine with odds ratios and confidence intervals

results <- cbind(

Odds_Ratio = exp(coef(logit_modd3)),

SE = coefs[, "Std. Error"],

z_value = coefs[, "z value"],

p_value = coefs[, "Pr(>|z|)"],

Lower_95CI = exp(confint(logit_modd3))[, 1],

Upper_95CI = exp(confint(logit_modd3))[, 2]

)

# View tidy table

round(results, 3)

logit_modd4 <- glm(NotWTP ~ Gender + Age_continuous_num + Income_continuous_num + AdultHHsize_cont +

FoodInsecurityIndex + FoodInsecurityIndex12,

data = WTPdataR, family = binomial)

summary(logit_modd4)

coefs <- summary(logit_modd4)$coefficients

# Combine with odds ratios and confidence intervals

results <- cbind(

Odds_Ratio = exp(coef(logit_modd4)),

SE = coefs[, "Std. Error"],

z_value = coefs[, "z value"],

p_value = coefs[, "Pr(>|z|)"],

Lower_95CI = exp(confint(logit_modd4))[, 1],

Upper_95CI = exp(confint(logit_modd4))[, 2]

)

# View tidy table

round(results, 3)

logit_modd5 <- glm(NotWTP ~ Gender + Age_continuous_num + Income_continuous_num + AdultHHsize_cont +

FoodInsecurityIndex + FoodInsecurityIndex12 + Perceptionofschlunch_3cat +

Typicallyeatlunch + Attendschbreakfastclub + Working + Ethnicity,

data = WTPdataR, family = binomial)

summary(logit_modd5)

coefs <- summary(logit_modd5)$coefficients

# Combine with odds ratios and confidence intervals

results <- cbind(

Odds_Ratio = exp(coef(logit_modd5)),

SE = coefs[, "Std. Error"],

z_value = coefs[, "z value"],

p_value = coefs[, "Pr(>|z|)"],

Lower_95CI = exp(confint(logit_modd5))[, 1],

Upper_95CI = exp(confint(logit_modd5))[, 2]

)

# View tidy table

round(results, 3)

library(pscl)

pR2(logit_modd3)

anova(logit_modd5, test = "Chisq")

library(sjPlot)

tab_model(logit_modd2, logit_modd3, logit_modd4, logit_modd5,

transform = "exp",

show.ci = TRUE,

show.se = TRUE,

p.style = "stars",

dv.labels = c("Model 2", "Model 3", "Model 4", "Model 5"))

library(stargazer)

# Compute odds ratios and robust SEs for all three models

logit_modd3_OR <- logit_modd3

logit_modd4_OR <- logit_modd4

logit_modd5_OR <- logit_modd5

# Replace coefficients with odds ratios

logit_modd3_OR$coefficients <- exp(coef(logit_modd3))

logit_modd4_OR$coefficients <- exp(coef(logit_modd4))

logit_modd5_OR$coefficients <- exp(coef(logit_modd5))

summ

# Create the table

stargazer(logit_modd3_OR, logit_modd4_OR, logit_modd5_OR,

type = "latex",

title = "Predictors of Parents’ Unwillingness to Co-Pay for School Lunches",

dep.var.labels = "Unwilling to Co-Pay (1 = No, 0 = Yes)",

covariate.labels = c("Female", "Age (years)", "Income", "Adult HH Size",

"Food Insecurity (1-Month)", "Food Insecurity (12-Month)",

"Perception of School Lunch", "Typically Eats School Lunch",

"Attends Breakfast Club", "Working", "Ethnicity (Minority)", "Gender * Income"),

ci = FALSE,

p.auto = TRUE,

se = list(

summary(logit_modd3)$coefficients[, "Std. Error"],

summary(logit_modd4)$coefficients[, "Std. Error"],

summary(logit_modd5)$coefficients[, "Std. Error"]

),

no.space = TRUE,

digits = 3,

star.cutoffs = c(0.1, 0.05, 0.01, 0.001),

notes = c("Odds Ratios reported.",

"Robust SEs in parentheses.",

" p < 0.1; * p < 0.05; ** p < 0.01; *** p < 0.001"))

# Example 1: Income × Household Size

logit_test1 <- glm(NotWTP ~ Gender + Age_continuous_num +

Income_continuous_num * AdultHHsize_cont +

FoodInsecurityIndex + FoodInsecurityIndex12 +

Perceptionofschlunch_3cat + Typicallyeatlunch +

Attendschbreakfastclub + Working + Ethnicity,

data = WTPdataR, family = binomial)

summary(logit_test1)

# Example 2: Perception × Typically Eats Lunch

logit_test2 <- glm(NotWTP ~ Gender + Age_continuous_num + Income_continuous_num +

AdultHHsize_cont + FoodInsecurityIndex + FoodInsecurityIndex12 +

Perceptionofschlunch_3cat * Typicallyeatlunch +

Attendschbreakfastclub + Working + Ethnicity,

data = WTPdataR, family = binomial)

summary(logit_test2)

logit_test3 <- glm(NotWTP ~ Gender + Age_continuous_num + Income_continuous_num * FoodInsecurityIndex +

AdultHHsize_cont + FoodInsecurityIndex12 +

Perceptionofschlunch_3cat + Typicallyeatlunch +

Attendschbreakfastclub + Working + Ethnicity,

data = WTPdataR, family = binomial)

summary(logit_test3)

logit_test4 <- glm(NotWTP ~ Gender + Age_continuous_num + Income_continuous_num * FoodInsecurityIndex12 +

AdultHHsize_cont + FoodInsecurityIndex +

Perceptionofschlunch_3cat + Typicallyeatlunch +

Attendschbreakfastclub + Working + Ethnicity,

data = WTPdataR, family = binomial)

summary(logit_test4)

logit_test5 <- glm(NotWTP ~ Gender * Income_continuous_num + Age_continuous_num +

AdultHHsize_cont +

FoodInsecurityIndex + FoodInsecurityIndex12 +

Perceptionofschlunch_3cat + Typicallyeatlunch +

Attendschbreakfastclub + Working + Ethnicity,

data = WTPdataR, family = binomial)

summary(logit_test5)

coefs <- summary(logit_test5)$coefficients

# Combine with odds ratios and confidence intervals

results <- cbind(

Odds_Ratio = exp(coef(logit_test5)),

SE = coefs[, "Std. Error"],

z_value = coefs[, "z value"],

p_value = coefs[, "Pr(>|z|)"],

Lower_95CI = exp(confint(logit_test5))[, 1],

Upper_95CI = exp(confint(logit_test5))[, 2]

)

# View tidy table

round(results, 3)

AIC(logit_test5)

logit_test6 <- glm(NotWTP ~ Gender + Income_continuous_num * Ethnicity + Age_continuous_num +

AdultHHsize_cont +

FoodInsecurityIndex + FoodInsecurityIndex12 +

Perceptionofschlunch_3cat + Typicallyeatlunch +

Attendschbreakfastclub + Working,

data = WTPdataR, family = binomial)

summary(logit_test6)

logit_test7 <- glm(NotWTP ~ Gender * Income_continuous_num + Age_continuous_num +

AdultHHsize_cont +

FoodInsecurityIndex + FoodInsecurityIndex12 * Working +

Perceptionofschlunch_3cat + Typicallyeatlunch +

Attendschbreakfastclub + Ethnicity,

data = WTPdataR, family = binomial)

summary(logit_test7)

logit_test8 <- glm(NotWTP ~ Gender + Income_continuous_num + Age_continuous_num +

AdultHHsize_cont +

FoodInsecurityIndex + FoodInsecurityIndex12 +

Perceptionofschlunch_3cat * Attendschbreakfastclub + Typicallyeatlunch + Working + Ethnicity,

data = WTPdataR, family = binomial)

summary(logit_test8)

logit_test5_OR <- logit_test5

logit_test5_OR$coefficients <- exp(coef(logit_test5))

stargazer(logit_modd3_OR, logit_modd4_OR, logit_modd5_OR, logit_test5_OR,

type = "latex",

title = "Predictors of Parents’ Unwillingness to Co-Pay for School Lunches",

dep.var.labels = "Unwilling to Co-Pay (1 = No, 0 = Yes)",

covariate.labels = c("Female", "Age (years)", "Income", "Adult HH Size",

"Food Insecurity (1-Month)", "Food Insecurity (12-Month)",

"Perception of School Lunch", "Typically Eats School Lunch",

"Attends Breakfast Club", "Working", "Ethnicity (Minority)", "Gender * Income"),

ci = FALSE,

p.auto = TRUE,

se = list(

summary(logit_modd3)$coefficients[, "Std. Error"],

summary(logit_modd4)$coefficients[, "Std. Error"],

summary(logit_modd5)$coefficients[, "Std. Error"],

summary(logit_test5)$coefficients[, "Std. Error"]

),

no.space = TRUE,

digits = 3,

star.cutoffs = c(0.1, 0.05, 0.01, 0.001),

notes = c("Odds Ratios reported.",

"Robust SEs in parentheses.",

" p < 0.1; * p < 0.05; ** p < 0.01; *** p < 0.001"))

# Load necessary packages

library(lmtest)

library(sandwich)

# --- For Model 24 ---

# Compute robust (HC1) variance–covariance matrix

robust_se_mod24 <- vcovHC(mod24, type = "HC1")

# Generate coefficient table with robust SEs

robust_test_mod24 <- coeftest(mod24, vcov = robust_se_mod24)

# Compute 95% confidence intervals

confint_mod24 <- coefci(mod24, vcov. = robust_se_mod24, level = 0.95)

# Combine results into a single table

results_mod24 <- cbind(

Estimate = coef(mod24),

"Robust SE" = sqrt(diag(robust_se_mod24)),

"t value" = robust_test_mod24[, "t value"],

"p value" = robust_test_mod24[, "Pr(>|t|)"],

confint_mod24

)

# View Model 24 results

round(results_mod24, 3)

# --- For Model 13 ---

# Compute robust (HC1) variance–covariance matrix

robust_se_mod_13 <- vcovHC(mod_13, type = "HC1")

# Generate coefficient table with robust SEs

robust_test_mod_13 <- coeftest(mod_13, vcov = robust_se_mod_13)

# Compute 95% confidence intervals

confint_mod_13 <- coefci(mod_13, vcov. = robust_se_mod_13, level = 0.95)

# Combine results into a single table

results_mod_13 <- cbind(

Estimate = coef(mod_13),

"Robust SE" = sqrt(diag(robust_se_mod_13)),

"t value" = robust_test_mod_13[, "t value"],

"p value" = robust_test_mod_13[, "Pr(>|t|)"],

confint_mod_13

)

# View Model 13 results

round(results_mod_13, 3)

# Assuming your data frame is called WTPdataR and has WTP1 and WTP2 columns

library(dplyr)

library(tidyr)

library(ggplot2)

# Define a price range

price_points <- seq(0, 5, by = 0.25)

# Function to compute demand (proportion willing to pay >= price)

get_demand <- function(wtp) {

sapply(price_points, function(p) mean(wtp >= p, na.rm = TRUE))

}

# Compute cumulative proportions for both WTP variables

demand_data <- data.frame(

Price = price_points,

Demand_WTP1 = get_demand(WTPdataR$WTP.1),

Demand_WTP2 = get_demand(WTPdataR$WTP.2)

)

# Reshape for ggplot

demand_long <- demand_data %>%

pivot_longer(cols = starts_with("Demand_"),

names_to = "Meal_Type",

values_to = "Demand") %>%

mutate(Meal_Type = recode(Meal_Type,

"Demand_WTP1" = "Standard Lunch",

"Demand_WTP2" = "Nutritious Lunch"))

# Add approximate 95% CIs for proportions

demand_long <- demand_long %>%

group_by(Meal_Type) %>%

mutate(

n = sum(!is.na(WTPdataR$WTP.1)), # total respondents with valid data

SE = sqrt((Demand * (1 - Demand)) / n),

CI_low = pmax(0, Demand - 1.96 * SE),

CI_high = pmin(1, Demand + 1.96 * SE)

)

# Plot demand curve

ggplot(demand_long, aes(x = Price, y = Demand, colour = Meal_Type)) +

geom_line(size = 1.2) +

geom_point(size = 2) +

geom_errorbar(aes(ymin = CI_low, ymax = CI_high), width = 0.05, alpha = 0.5) +

scale_y_continuous(labels = scales::percent_format(accuracy = 1)) +

scale_colour_manual(values = c("#E69F00", "#0072B2")) +

labs(

title = "Demand Curve for School Meals",

x = "Willingness to Pay (£)",

y = "Proportion of Respondents (%)",

colour = "Meal Type"

) +

theme_minimal(base_size = 14) +

theme(

plot.title = element_text(face = "bold", hjust = 0.5),

legend.position = "bottom"

)

# Compute marginal effects (average across all observations)

margins_tobit1 <- margins(tobit1)

summary(margins_tobit1)

coefs <- coef(tobit1)

sigma <- tobit1$scale

coefs

sigma

library(AER)

library(dplyr)

tobit1 <- AER::tobit(

WTP.1 ~ Income_continuous_num + Age_continuous_num + Gender + Ethnicity +

AdultHHsize_cont + FoodInsecurityIndex + FoodInsecurityIndex12 +

Perceptionofschlunch_3cat + Typicallyeatlunch +

Attendschbreakfastclub + Working,

data = WTPdataR,

left = 0, right = 6

)

coefs <- coef(tobit1)

sigma <- tobit1$scale

print(coefs)

print(sigma)

# Predicted linear term

xb <- predict(tobit1, type = "link")

# Compute z and probabilities

z <- xb / sigma

Phi_z <- pnorm(z)

# Average probability of being uncensored

p_uncensored <- mean(Phi_z)

# Marginal effects

marginal_effects <- coefs * p_uncensored

# Tidy table

marginal_effects_table <- data.frame(

Variable = names(coefs),

Coefficient = round(coefs, 3),

Marginal_Effect = round(marginal_effects, 3)

)

marginal_effects_table

tobit2 <- AER::tobit(

WTP.2 ~ Income_continuous_num + Age_continuous_num + Gender + Ethnicity +

AdultHHsize_cont + FoodInsecurityIndex + FoodInsecurityIndex12 +

Perceptionofschlunch_3cat + Typicallyeatlunch +

Attendschbreakfastclub + Working,

data = WTPdataR,

left = 0, right = 6

)

# Predicted linear term and sigma

xb2 <- predict(tobit2, type = "link")

sigma2 <- tobit2$scale

# Compute Φ(z)

z2 <- xb2 / sigma2

Phi_z2 <- pnorm(z2)

p_uncensored2 <- mean(Phi_z2)

# Marginal effects

coefs2 <- coef(tobit2)

marginal_effects2 <- coefs2 * p_uncensored2

# Tidy table

marginal_effects_table2 <- data.frame(

Variable = names(coefs2),

Coefficient = round(coefs2, 3),

Marginal_Effect = round(marginal_effects2, 3)

)

marginal_effects_table2

# Extract only coefficient names (excluding sigma)

coefs_tobit2 <- tobit2$coefficients

vcov_tobit2 <- tobit2$var[1:length(coefs_tobit2), 1:length(coefs_tobit2)]

# Compute standard errors

se_tobit2 <- sqrt(diag(vcov_tobit2))

# Compute z-values and p-values

z_tobit2 <- coefs_tobit2 / se_tobit2

p_tobit2 <- 2 * (1 - pnorm(abs(z_tobit2)))

# Compute 95% CI

ci_lower_tobit2 <- coefs_tobit2 - 1.96 * se_tobit2

ci_upper_tobit2 <- coefs_tobit2 + 1.96 * se_tobit2

# Combine into one clean table

tobit2_results <- data.frame(

Variable = names(coefs_tobit2),

Estimate = round(coefs_tobit2, 3),

Std_Error = round(se_tobit2, 3),

z_value = round(z_tobit2, 3),

p_value = round(p_tobit2, 3),

CI_lower = round(ci_lower_tobit2, 3),

CI_upper = round(ci_upper_tobit2, 3)

)

tobit2_results

#Tobit 1 co-effecients, SE, P-value and 95% CI

coefs_tobit1 <- tobit1$coefficients

vcov_tobit1 <- tobit1$var[1:length(coefs_tobit1), 1:length(coefs_tobit1)]

# Compute standard errors

se_tobit1 <- sqrt(diag(vcov_tobit1))

# Compute z-values and p-values

z_tobit1 <- coefs_tobit1 / se_tobit1

p_tobit1 <- 2 * (1 - pnorm(abs(z_tobit1)))

# Compute 95% CI

ci_lower_tobit1 <- coefs_tobit1 - 1.96 * se_tobit1

ci_upper_tobit1 <- coefs_tobit1 + 1.96 * se_tobit1

# Combine into one clean table

tobit1_results <- data.frame(

Variable = names(coefs_tobit1),

Estimate = round(coefs_tobit1, 3),

Std_Error = round(se_tobit1, 3),

z_value = round(z_tobit1, 3),

p_value = round(p_tobit1, 3),

CI_lower = round(ci_lower_tobit1, 3),

CI_upper = round(ci_upper_tobit1, 3)

)

tobit1_results

tobit1_int <- AER::tobit(

WTP.1 ~ Income_continuous_num * AdultHHsize_cont +

Perceptionofschlunch_3cat * Typicallyeatlunch +

Age_continuous_num + Ethnicity + FoodInsecurityIndex + FoodInsecurityIndex12 +

Attendschbreakfastclub + Working,

data = WTPdataR, left = 0, right = 6

)

# --- FOR WTP1 INTERACTION MODEL ---

coefs_tobit1_int <- tobit1_int$coefficients

vcov_tobit1_int <- tobit1_int$var[1:length(coefs_tobit1_int), 1:length(coefs_tobit1_int)]

# Compute standard errors

se_tobit1_int <- sqrt(diag(vcov_tobit1_int))

# Compute z-values and p-values

z_tobit1_int <- coefs_tobit1_int / se_tobit1_int

p_tobit1_int <- 2 * (1 - pnorm(abs(z_tobit1_int)))

# Compute 95% confidence intervals

ci_lower_tobit1_int <- coefs_tobit1_int - 1.96 * se_tobit1_int

ci_upper_tobit1_int <- coefs_tobit1_int + 1.96 * se_tobit1_int

# Combine into clean table

tobit1_int_results <- data.frame(

Variable = names(coefs_tobit1_int),

Estimate = round(coefs_tobit1_int, 3),

Std_Error = round(se_tobit1_int, 3),

z_value = round(z_tobit1_int, 3),

p_value = round(p_tobit1_int, 3),

CI_lower = round(ci_lower_tobit1_int, 3),

CI_upper = round(ci_upper_tobit1_int, 3)

)

tobit1_int_results

AIC(tobit1, tobit1_int)

tobit2_int <- AER::tobit(

WTP.2 ~ Income_continuous_num * AdultHHsize_cont +

Perceptionofschlunch_3cat * Typicallyeatlunch +

Age_continuous_num + Ethnicity + FoodInsecurityIndex + FoodInsecurityIndex12 +

Attendschbreakfastclub + Working,

data = WTPdataR, left = 0, right = 6

)

# --- FOR WTP2 INTERACTION MODEL ---

coefs_tobit2_int <- tobit2_int$coefficients

vcov_tobit2_int <- tobit2_int$var[1:length(coefs_tobit2_int), 1:length(coefs_tobit2_int)]

se_tobit2_int <- sqrt(diag(vcov_tobit2_int))

z_tobit2_int <- coefs_tobit2_int / se_tobit2_int

p_tobit2_int <- 2 * (1 - pnorm(abs(z_tobit2_int)))

ci_lower_tobit2_int <- coefs_tobit2_int - 1.96 * se_tobit2_int

ci_upper_tobit2_int <- coefs_tobit2_int + 1.96 * se_tobit2_int

tobit2_int_results <- data.frame(

Variable = names(coefs_tobit2_int),

Estimate = round(coefs_tobit2_int, 3),

Std_Error = round(se_tobit2_int, 3),

z_value = round(z_tobit2_int, 3),

p_value = round(p_tobit2_int, 3),

CI_lower = round(ci_lower_tobit2_int, 3),

CI_upper = round(ci_upper_tobit2_int, 3)

)

tobit2_int_results

AIC(tobit2, tobit2_int)
